# Composing recurrent spiking neural networks using locally-recurrent motifs and risk-mitigating architectural optimization

**DOI:** 10.3389/fnins.2024.1412559

**Published:** 2024-06-20

**Authors:** Wenrui Zhang, Hejia Geng, Peng Li

**Affiliations:** Department of Electrical and Computer Engineering, University of California, Santa Barbara, Santa Barbara, CA, United States

**Keywords:** brain inspired computing, recurrent spiking neural networks, neural architecture search, Sparsely-Connected Recurrent Motif Layer, intrinsic plasticity

## Abstract

In neural circuits, recurrent connectivity plays a crucial role in network function and stability. However, existing recurrent spiking neural networks (RSNNs) are often constructed by random connections without optimization. While RSNNs can produce rich dynamics that are critical for memory formation and learning, systemic architectural optimization of RSNNs is still an open challenge. We aim to enable systematic design of large RSNNs via a new scalable RSNN architecture and automated architectural optimization. We compose RSNNs based on a layer architecture called Sparsely-Connected Recurrent Motif Layer (SC-ML) that consists of multiple small recurrent motifs wired together by sparse lateral connections. The small size of the motifs and sparse inter-motif connectivity leads to an RSNN architecture scalable to large network sizes. We further propose a method called Hybrid Risk-Mitigating Architectural Search (HRMAS) to systematically optimize the topology of the proposed recurrent motifs and SC-ML layer architecture. HRMAS is an alternating two-step optimization process by which we mitigate the risk of network instability and performance degradation caused by architectural change by introducing a novel biologically-inspired “self-repairing” mechanism through intrinsic plasticity. The intrinsic plasticity is introduced to the second step of each HRMAS iteration and acts as unsupervised fast self-adaptation to structural and synaptic weight modifications introduced by the first step during the RSNN architectural “evolution.” We demonstrate that the proposed automatic architecture optimization leads to significant performance gains over existing manually designed RSNNs: we achieve 96.44% on TI46-Alpha, 94.66% on N-TIDIGITS, 90.28% on DVS-Gesture, and 98.72% on N-MNIST. To the best of the authors' knowledge, this is the first work to perform systematic architecture optimization on RSNNs.

## 1 Introduction

In the brain, recurrent connectivity is indispensable for maintaining dynamics, functions, and oscillations of the network (Buzsaki, 2006). As a brain-inspired computational model, spiking neural networks (SNNs) are well suited for processing spatiotemporal information (Maass, 1997). In particular, recurrent spiking neural networks (RSNNs) can mimic microcircuits in the biological brain and induce rich behaviors that are critical for memory formation and learning. Recurrence has been explored in conventional non-spiking artificial neural networks (ANNs) in terms of Long Short Term Memory (LSTM) (Hochreiter and Schmidhuber, 1997), Echo State Networks (ESN) (Jaeger, 2001), Deep RNNs (Graves et al., 2013), Gated Recurrent Units (GRU) (Cho et al., 2014), and Legendre Memory Units (LMU) (Voelker et al., 2019). While recurrence presents unique challenges and opportunities in the context of spiking neural networks, RSNNs are yet to be well explored.

Most existing works on RSNNs adopt recurrent layers or reservoirs with randomly generated connections. The Liquid State Machine (LSM) (Maass et al., 2002) is one of the most widely adopted RSNN architectures with one or multiple recurrent reservoirs and an output readout layer wired up using feedforward synapses (Zhang et al., 2015; Wang and Li, 2016; Srinivasan et al., 2018). However, there is a lack of principled approaches for setting up the recurrent connections in reservoirs. Instead, *ad-hoc* randomly generated wiring patterns are often adopted. Bellec et al. (2018) proposed an architecture called long short-term memory SNNs (LSNNs). The recurrent layer contains a regular spiking portion with both inhibitory and excitatory spiking neurons and an adaptive neural population. Zhang and Li (2019b) proposed to train deep RSNNs by a spike-train level backpropagation (BP) method. Maes et al. (2020) demonstrated a new reservoir with multiple groups of excitatory neurons and a central group of inhibitory neurons. Furthermore, Zhang and Li (2021b) presented a recurrent structure named ScSr-SNNs in which recurrence is simply formed by a self-recurrent connection to each neuron. However, the recurrent connections in all of these works are either randomly generated with certain probabilities or simply constructed by self-recurrent connections. Randomly generated or simple recurrent connections may not effectively optimize RSNNs' performance. Recently, Pan et al. (2024) introduced a multi-objective Evolutionary Liquid State Machine (ELSM) inspired by neuroevolution process. Chakraborty and Mukhopadhyay (2023) proposed Heterogeneous recurrent spiking neural network (HRSNN), in which recurrent layers are composed of heterogeneous neurons with different dynamics. Chen et al. (2023) introduced an intralayer-connected SNN and a hybrid training method combining probabilistic spike-timing dependent plasticity (STDP) with BP. But their performance still has significant gaps. Systemic RSNN architecture design and optimization remain as an open problem.

Neural architectural search (NAS), the process of automating the construction of non-spiking ANNs, has become prevalent recently after achieving state-of-the-art performance on various tasks (Elsken et al., 2019; Wistuba et al., 2019). Different types of strategies such as reinforcement learning (Zoph and Le, 2017), gradient-based optimization (Liu et al., 2018), and evolutionary algorithms (Real et al., 2019) have been proposed to find optimal architectures of traditional CNNs and RNNs. In contrast, the architectural optimization of SNNs has received little attention. Only recently, Tian et al. (2021) adopted a simulated annealing algorithm to learn the optimal architecture hyperparameters of liquid state machine (LSM) models through a three-step search. Similarly, a surrogate-assisted evolutionary search method was applied in Zhou et al. (2020) to optimize the hyperparameters of LSM such as density, probability and distribution of connections. However, both studies focused only on LSM for which hyperparameters indirectly affecting recurrent connections as opposed to specific connectivity patterns were optimized. Even after selecting the hyperparameters, the recurrence in the network remained randomly determined without any optimization. Recently, Kim et al. (2022) explored a cell-based neural architecture search method on SNNs, but did not involve large-scale recurrent connections. Na et al. (2022) introduced a spike-aware NAS framework called AutoSNN to investigate the impact of architectural components on SNNs' performance and energy efficiency. Overall, NAS for RSNNs is still rarely explored.

This paper aims to enable systematic design of large recurrent spiking neural networks (RSNNs) via a new scalable RSNN architecture and automated architectural optimization. RSNNs can create complex network dynamics both in time and space, which manifests itself as an opportunity for achieving great learning capabilities and a challenge in practical realization. It is important to strike a balance between theoretical computational power and architectural complexity. Firstly, we argue that composing RSNNs based on well-optimized building blocks small in size, or recurrent motifs, can lead to an architectural solution scalable to large networks while achieving high performance. We assemble multiple recurrent motifs into a layer architecture called Sparsely-Connected Recurrent Motif Layer (SC-ML). The motifs in each SC-ML share the same *topology*, defined by the size of the motif, i.e., the number of neurons, and the recurrent connectivity pattern between the neurons. The motif topology is determined by the proposed architectural optimization while the weights within each motif may be tuned by standard backpropagation training algorithms. Motifs in a recurrent SC-ML layer are wired together using sparse lateral connections determined by imposing spatial connectivity constraints. As such, there exist two levels of structured recurrence: recurrence within each motif and recurrence between the motifs at the SC-ML level. The fact that the motifs are small in size and that inter-motif connectivity is sparse alleviates the difficulty in architectural optimization and training of these motifs and SC-ML. Furthermore, multiple SC-ML layers can be stacked and wired using additional feedforward weights to construct even larger recurrent networks.

Secondly, we demonstrate a method called Hybrid Risk-Mitigating Architectural Search (HRMAS) to optimize the proposed recurrent motifs and SC-ML layer architecture. HRMAS is an alternating two-step optimization process hybridizing bio-inspired intrinsic plasticity for mitigating the risk in architectural optimization. Facilitated by gradient-based methods (Liu et al., 2018; Zhang and Li, 2020), the first step of optimization is formulated to optimize network architecture defined by the size of the motif, intra and inter-motif connectivity patterns, types of these connections, and the corresponding synaptic weight values, respectively.

While structural changes induced by the architectural-level optimization are essential for finding high-performance RSNNs, they may be misguided due to discontinuity in architectural search, and limited training data, hence leading to over-fitting. We mitigate the risk of network instability and performance degradation caused by architectural change by introducing a novel biologically-inspired “self-repairing” mechanism through intrinsic plasticity, which has the same spirit of homeostasis during neural development (Tien and Kerschensteiner, 2018). The intrinsic plasticity is introduced in the second step of each HRMAS iteration and acts as unsupervised self-adaptation to mitigate the risks imposed by structural and synaptic weight modifications introduced by the first step during the RSNN architectural “evolution.”

We evaluate the proposed techniques on speech dataset TI46-Alpha (Liberman et al., 1991), neuromorphic speech dataset N-TIDIGITS (Anumula et al., 2018), neuromorphic video dataset DVS-Gesture (Amir et al., 2017), and neuromorphic image dataset N-MNIST (Orchard et al., 2015). The SC-ML-based RSNNs optimized by HRMAS achieve state-of-the-art performance on all four datasets. With the same network size, automated network design via HRMAS outperforms existing RSNNs by up to 3.38% performance improvement.

## 2 Materials and methods

### 2.1 Spiking neuron model

In this work, we adopt the leaky integrate-and-fire (LIF) neuron model Gerstner and Kistler (2002) which is one of the most popular neuron models for simulating SNNs. During the simulation, we use the fixed-step first-order Euler method to discretize the LIF model. In the rest of this paper, we only analyze an SNN in the discretized form. Consider the input spike train from pre-synaptic neuron *j*: $s_{j}\left[t\right]=\underset{t_{j}^{\left(f\right)}}{\sum }\delta \left[t-t_{j}^{\left(f\right)}\right]$, where $t_{j}^{\left(f\right)}$ denotes a particular firing time of presynaptic neuron *j*. The incoming spikes are converted into an (unweighted) postsynaptic current (PSC) *a*_*j*_[*t*] through a synaptic model. We adopt the first-order synaptic model in Equation (1): (Gerstner and Kistler, 2002):


(1) $$\begin{array}{l} a_{j}\left[t\right]=\left(1-\frac{1}{\tau_{syn}}\right)a_{j}\left[t-1\right]+s_{j}\left[t\right], \end{array}$$

where τ_*syn*_ is the synaptic time constant. Then, the neuronal membrane voltage *u*_*i*_[*t*] of neuron *i* at time *t* is given in Equation (2) and Equation (3):


(2) $$\begin{array}{l} u_{i}^{-}\left[t\right]=\left(1-\frac{1}{\tau }\right)u_{i}\left[t-1\right]+\frac{R}{\tau }\underset{j}{\sum }w_{ij}a_{j}\left[t\right], \end{array}$$

(3) $$\begin{array}{l} u_{i}\left[t\right]=\{\begin{array}{ll} 0, & \text{if }u_{i}^{-}\left[t\right]>V_{\text{th}}\\ u_{i}^{-}\left[t\right], & \text{otherwise} \end{array} \end{array}$$

where *R* and τ are the resistance and time constant of the membrane, *w*_*ij*_ the synaptic weight from pre-synaptic neuron *j* to neuron *i*. Moreover, the firing output of the neuron is expressed in Equation (4)


(4) $$\begin{array}{l} s_{i}\left[t\right]=H\left(u_{i}^{-}\left[t\right]-V_{th}\right) \end{array}$$

where *V*_*th*_ is the firing threshold and *H*(·) is the Heaviside step function.

### 2.2 Sparsely-Connected Recurrent Motif Layer

**Figure 1:**
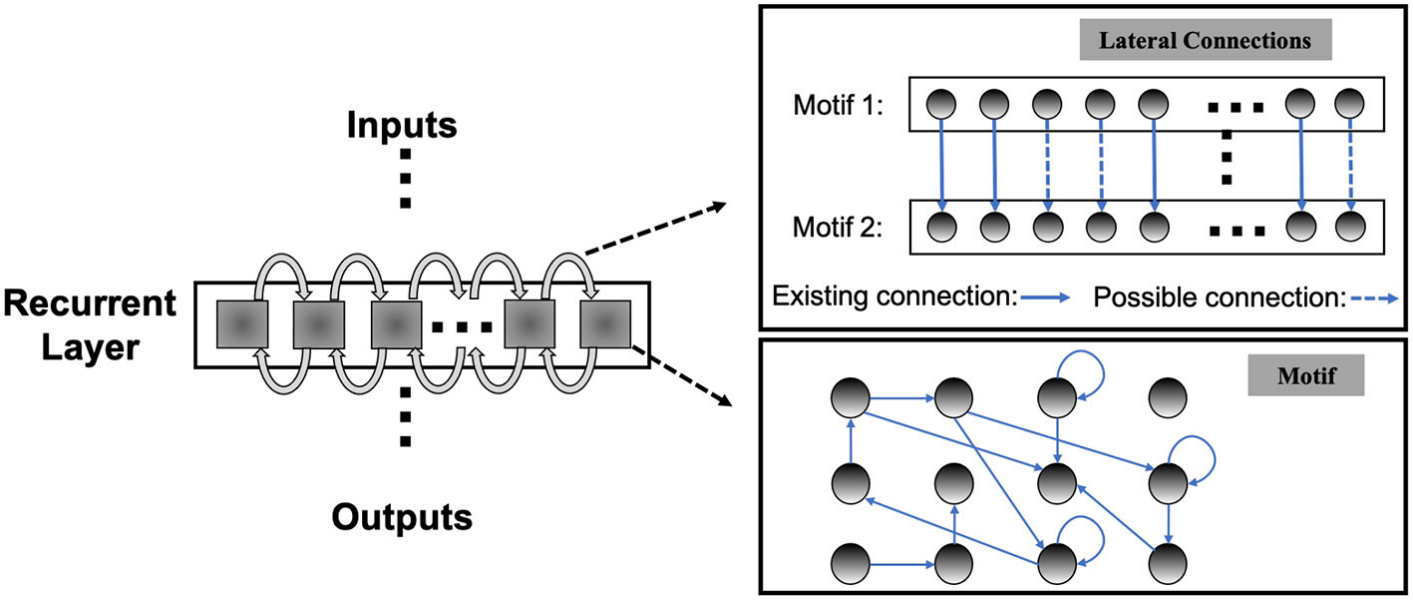
Sparsely-connected recurrent motif layer.

Unlike the traditional non-spiking RNNs that are typically constructed with units like LSTM or GRU, the structure of existing RSNNs is random without specific optimization, which hinders RSNNs' performance and prevents scaling to large networks. However, due to the complexity of recurrent connections and dynamics of spiking neurons, the optimization of RSNNs weights is still an open problem. As shown in **Table 3**, recurrent connections that are not carefully set up may hinder network performance. To solve this problem, we first designed the SC-ML layer, which is composed of multiple sparsely-connected recurrent *motifs*, where each motif consists of a group of recurrently connected spiking neurons, as shown in Figure 1. The motifs in each SC-ML share the same topology, which is defined as the size of the motif, i.e., the number of neurons, and the recurrent connectivity pattern between the neurons (excitatory, inhibitory or non-existent). Within the motif, synaptic connections can be constructed between any two neurons including self-recurrent connections. Thus the problem of the recurrent layer optimization can be simplified to that of learning the optimal motif and sparse inter-motif connectivity, alleviating the difficulty in architectural optimization and allowing scalability to large networks.

This motif-based structure is motivated by both a biological and a computational perspective. First, from a biological point of view, there is evidence that the neocortex is not only organized in layered minicolumn structures but also into synaptically connected clusters of neurons within such structures (Ko et al., 2011; Perin et al., 2011). For example, the networks of pyramidal cells cluster into multiple groups of a few dozen neurons each. Second, we add onto the memory effects resulting from temporal integration of individual spiking neurons by introducing sparse intra or inter-motif connections. This corresponds to a scalable and biologically plausible RSNN architectural design space that closely mimics the microcircuits in the nervous system. From a computational perspective, optimizing the connectivity of the basic building block, i.e., the motif, simplifies the problem of optimizing the connectivity of the whole recurrent layer. Furthermore, by constraining most recurrent connections inside the motifs and allowing a few lateral connections between neighboring motifs to exchange information across the SC-ML, the total number of recurrent connections is limited. This leads to a great deal of sparsity as observed in biological networks (Seeman et al., 2018).

Figure 1 presents an example of SC-ML with 12-neuron motifs. The lateral inter-motif connections can be introduced as the mutual connections between two corresponding neurons in neighboring motifs to ensure sparsity and reduce complexity. With the proposed SC-ML, one can easily stack multiple SC-MLs to form a multi-layer large RSNN using feedforward weights. Within a multi-layered network, information processing is facilitated through local processing of different motifs, communication of motif-level responses via inter-motif connections, and extraction and processing of higher-level features layer by layer.

### 2.3 Hybrid risk-mitigating architectural search

Neural architecture search (NAS) has been applied for architectural optimization of traditional non-spiking RNNs, where a substructure called cell is optimized by a search algorithm (Zoph and Le, 2017). Nevertheless, this NAS approach may not be the best fit for RSNNs. First, recurrence in the cell is only created by feeding previous hidden state back to the cell while connectivity inside the cell is feedforward. Second, the overall operations and connectivity found by the above NAS procedure do not go beyond an LSTM-like architecture. Finally, the considered combination operations and activation functions like addition and elementwise multiplication are not biologically plausible.

In order to extend NAS to a wider range of spiking RNNs, we introduce the Hybrid Risk-Mitigating Architectural Search (HRMAS). This framework systematically optimizes the motif topology and lateral connections of SC-ML. Each optimization iteration consists of two alternating steps, hybridizing gradient-based optimization and biologically-inspired intrinsic plasticity for robust NAS of RSNNs. We will introduce the overall idea of HRMAS in 2.3.1, the optimization problem of HRMAS in 2.3.2, the gradient-based optimization part in 2.3.3, and the bio-inspired optimization part in 2.3.4.

#### 2.3.1 Hybrid risk-mitigating architectural search framework

In HRMAS, all recurrent connections are categorized into three types: inhibitory, excitatory, and non-existence. An inhibitory connection has a negative weight and is fixed without training in our current implementation. In the recurrent network, negative weights mainly provide the function of inhibitory stimulation. Here we follow the settings in previous research (Zhang and Li, 2021b,a) and adopt fixed negative weights. In experiments, fixed negative weights can reduce the optimization complexity without significant performance loss, while providing stable inhibitory connections. The weight of an excitatory connection is positive and trained by a backpropagation (BP) method. HRMAS is an alternating two-step optimization process, hybridizing architectural optimization with intrinsic plasticity (IP). The first step of each HRMAS optimization iteration optimizes the topology of the motif and inter-motif connectivity in SC-ML and the corresponding synaptic weights hierarchically. Specifically, the optimal number of neurons in the motif is optimized over a finite set of motif sizes. All possible intra-motif connections are considered and the type of each connection is optimized, which may lead to a sparser connectivity if the connection types of certain synapses are determined to be “non-existence.” At the inter-motif level, a sparse motif-to-motif connectivity constraint is imposed: neurons in one motif are only allowed to be wired up with the corresponding neurons in the neighboring motifs as the Figure 1 shows. This locally connected topology will serve as a hard constraint in the subsequent optimization process. Inter-motif connections also fall under one of the three types (“inhibitory,” “excitatory,” “non-existence”). Hence, a greater level of sparsity is produced with the emergence of connections of type “non-existence.” The second step in each HRMAS iteration executes an unsupervised IP rule to stabilize the network function and mitigate potential risks caused by architectural changes.

**Figure 2:**
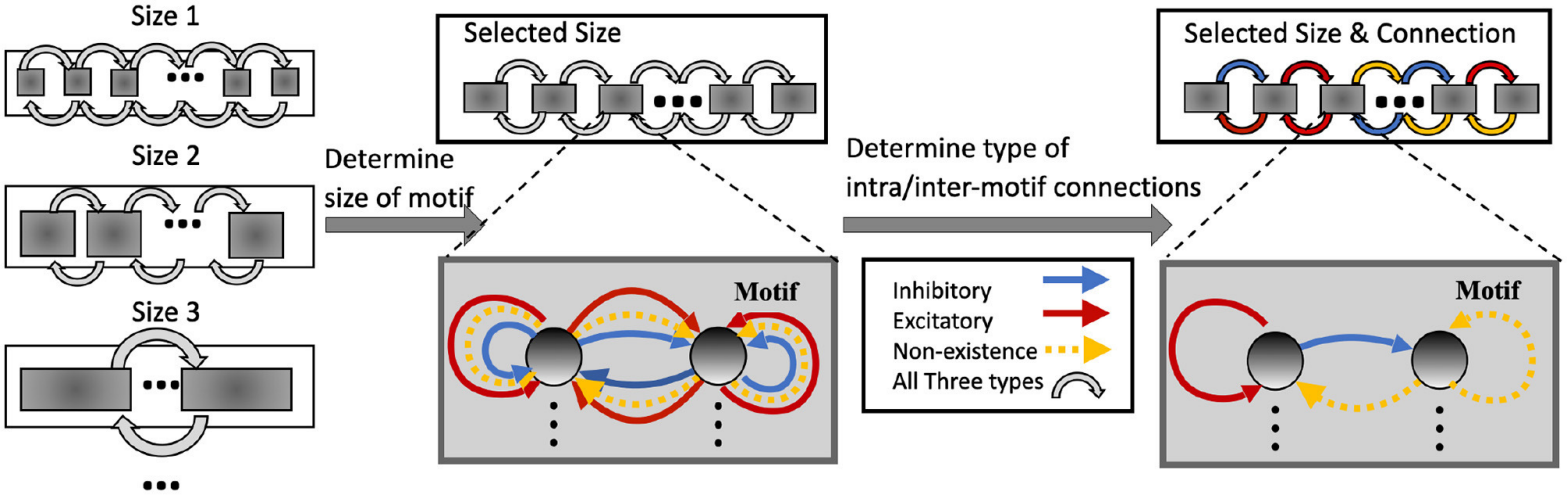
Architectural optimization in HRMAS.

Figure 2 illustrates the incremental optimization strategy we adopt for the architectural parameters. Using the two-step optimization, initially all architectural parameters including motif size and connectivity are optimized. After several training iterations, we choose the optimal motif size from a set of discrete options. As the most critical architectural parameter is set, we continue to optimize the remaining architectural parameters defining connectivity, allowing fine-tuning of performance based on the chosen motif size.

#### 2.3.2 Alternating two-step optimization in HRMAS

**Figure 3:**
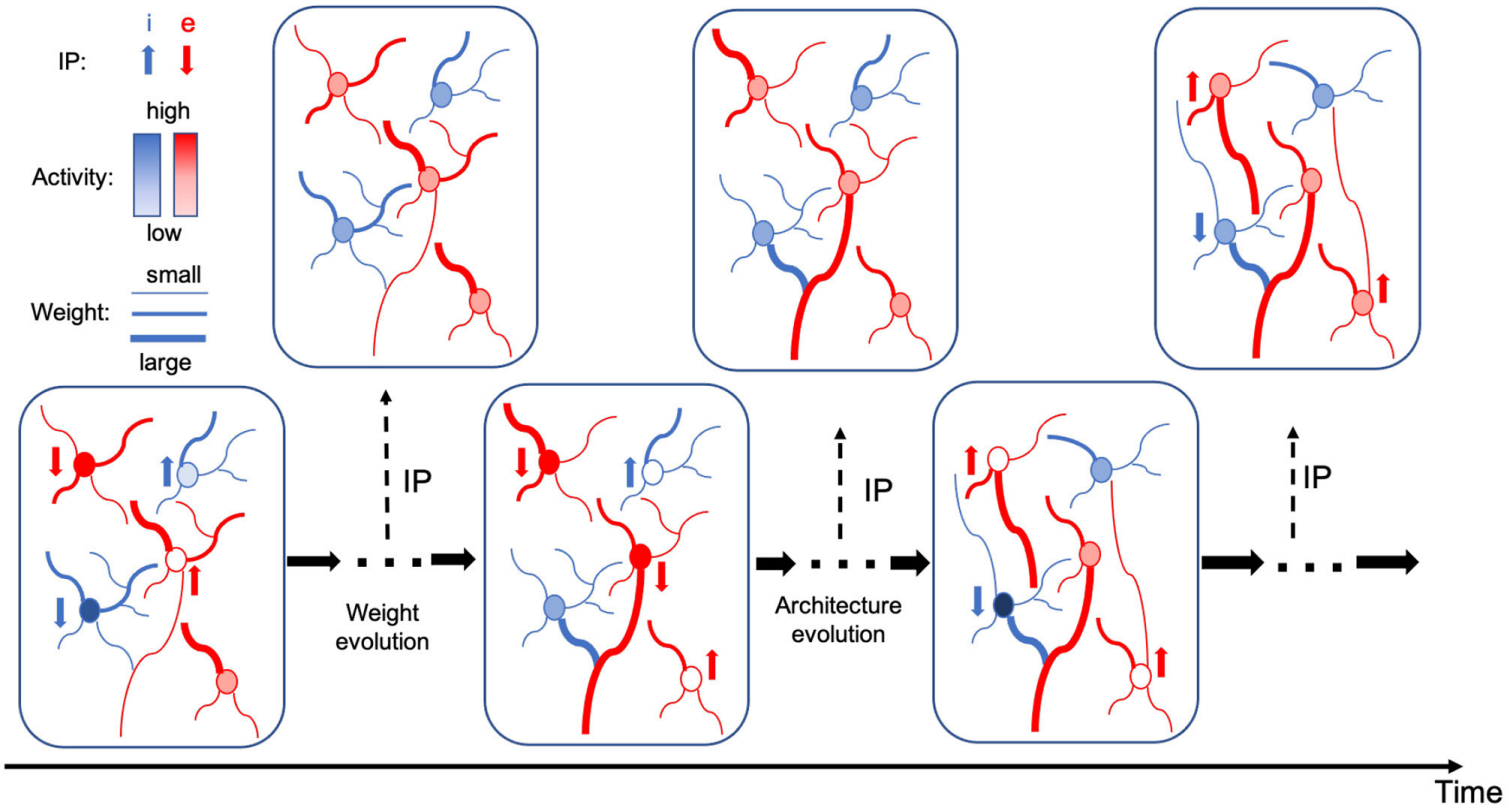
Evolution in neural development.

**Figure 4:**
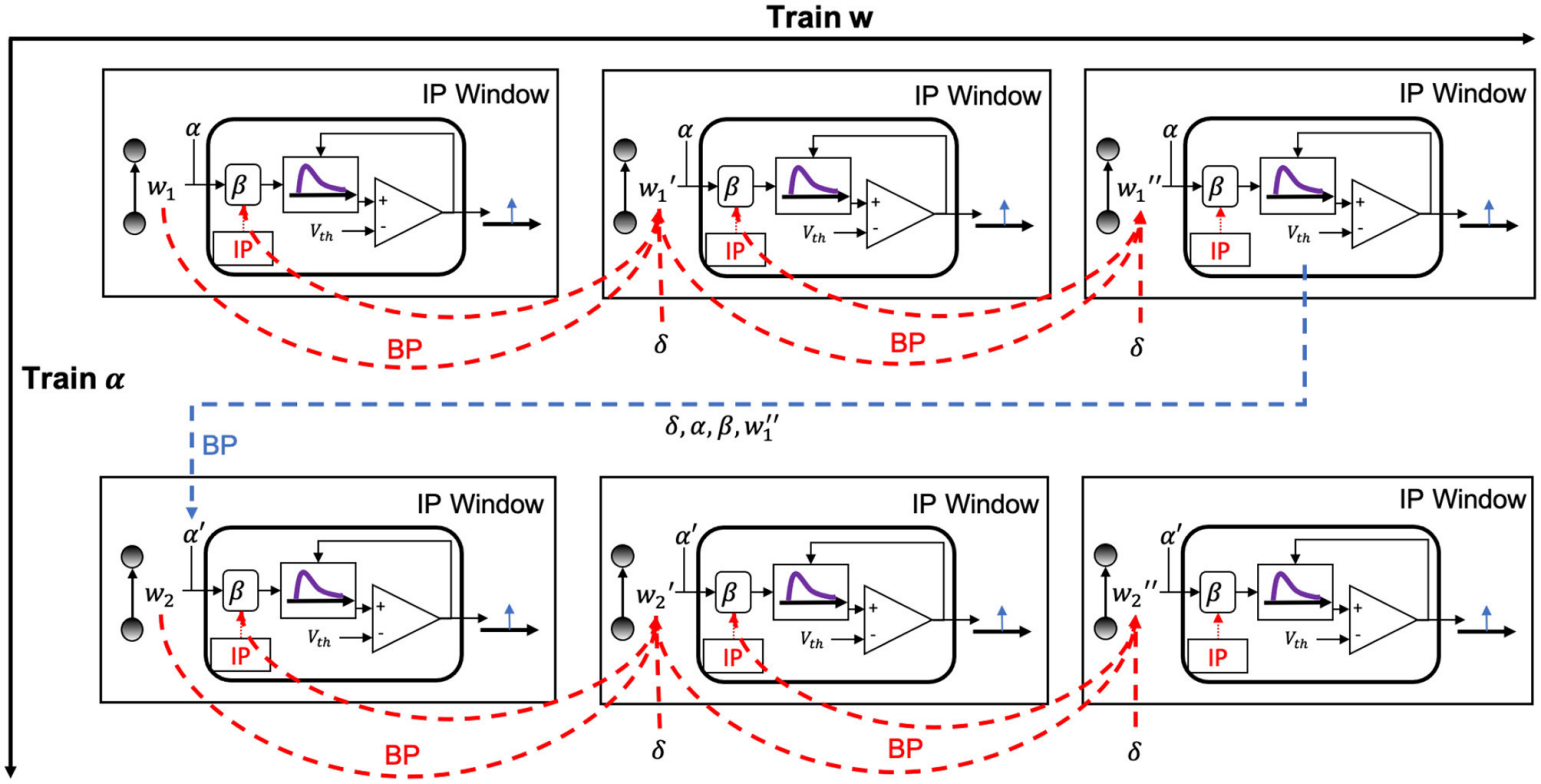
Proposed HRMAS.

The alternating two-step optimization in HRMAS is inspired by the evolution in neural development. As shown in Figure 3, neural circuits may experience weight changes through synaptic plasticity. Over a longer time scale, circuit architecture, i.e., connectivity, may evolve through learning and environmental changes. In addition, spontaneous firing behaviors of individual neurons may be adapted by intrinsic plasticity (IP). We are motivated by the important role of local IP mechanisms in stabilizing neuronal activity and coordinating structural changes to maintain proper circuit functions (Tien and Kerschensteiner, 2018). We view IP as a “fast-paced” self-adapting mechanism of individual neurons to react to and minimize the risks of weight and architectural modifications. As shown in Figure 4, we define the architectural parameters (motif size and intra/inter-motif connection types weights), synaptic weights, and intrinsic neuronal parameters as α, *w*, and β, respectively. Each HRMAS optimization iteration consists of two alternating steps. In the first step, we optimize α and *w* hierarchically based on gradient-based optimization using backpropagation (BP). In Figure 4, δ is the backpropagated error obtained via the employed BP method. In the second step, we use an unsupervised IP rule to adapt the intrinsic neuronal parameters of each neuron over a time window (“IP window”) during which training examples are presented to the network. IP allows the neurons to respond to the weight and architectural changes introduced in the first step and mitigate possible risks caused by such changes. In Step 1 of the subsequent iteration, the error gradients w.r.t the synaptic weights and architectural parameters are computed based on the most recent values of β updated in the preceding iteration. In summary, the *k*-th HRMAS iteration solves a bi-level optimization problem:


(5) $$\begin{array}{l} \alpha^{*}=\arg\underset{\alpha }{\min}L_{\text{valid}}\left(\alpha ,w^{*}\left(\alpha \right),\beta^{*}\right) \end{array}$$

(6) $$\begin{array}{l} \text{s.t. }\beta^{*}=\arg\underset{\beta }{\min}L_{\text{ip}}\left(\alpha ,w^{*}\left(\alpha \right),\beta_{-}^{*}\right), \end{array}$$

(7) $$\begin{array}{l} \text{s.t. }w^{*}\left(\alpha \right)=\arg\underset{w}{\min}L_{\text{train}}\left(\alpha ,w,\beta_{-}^{*}\right), \end{array}$$

where $L_{valid}$ and $L_{train}$ are the loss functions defined based on the validation and training sets used to train α and *w* respectively; $L_{ip}$ is the local loss to be minimized by the IP rule as further discussed in Section 2.3.4; $\beta_{-}^{*}$ are the intrinsic parameter values updated in the preceding (*k* − 1)-th iteration; w^*^(α) denotes the optimal synaptic weights under the architecture specified by α.

In the implementation of HRMAS, architectural parameters and synaptic weights are optimized by the first step. The architectural parameters are defined as motif size and types of intra/inter-motif connections. The general architectural optimization is performed by generating architecture and evaluating the architecture by a standard training and validation process on data. The validation performance is used to train the architectural parameters and generate a better structure. These steps are repeated until the optimal architecture is found. The first step of the *k*-th HRMAS iteration solves a bi-level optimization problem in Equations (8, 9) using BP:


(8) $$\begin{array}{l} min_{\alpha }L_{valid}\left(\alpha ,w^{*}\left(\alpha \right),\beta_{-}^{*}\right) \end{array}$$

(9) $$\begin{array}{l} s.t.\;w^{*}\left(\alpha \right)=arg_{w}minL_{train}\left(\alpha ,w,\beta_{-}^{*}\right), \end{array}$$

where $L_{valid}$ and $L_{train}$ are the loss functions defined based on the validation and training sets used to train α and *w* respectively; $\beta_{-}^{*}$ is the intrinsic parameter values updated in the preceding (*k* − 1)-th iteration; w^*^(α, β) denotes the optimal synaptic weights under the architecture specified by α. The second step of the *k*-th iteration solves the optimization problem Equation (10):


(10) $$\begin{array}{l} \beta^{*}=arg_{\beta }minL_{ip}\left(\alpha^{*},w^{*},\beta \right) \end{array}$$

$L_{ip}$ is the local loss to be minimized by the IP rule.

#### 2.3.3 Gradient-based optimization in HRMAS

##### 2.3.3.1 Relaxing SC-ML layer's architectural parameters from discrete to continuous

**Figure 5:**
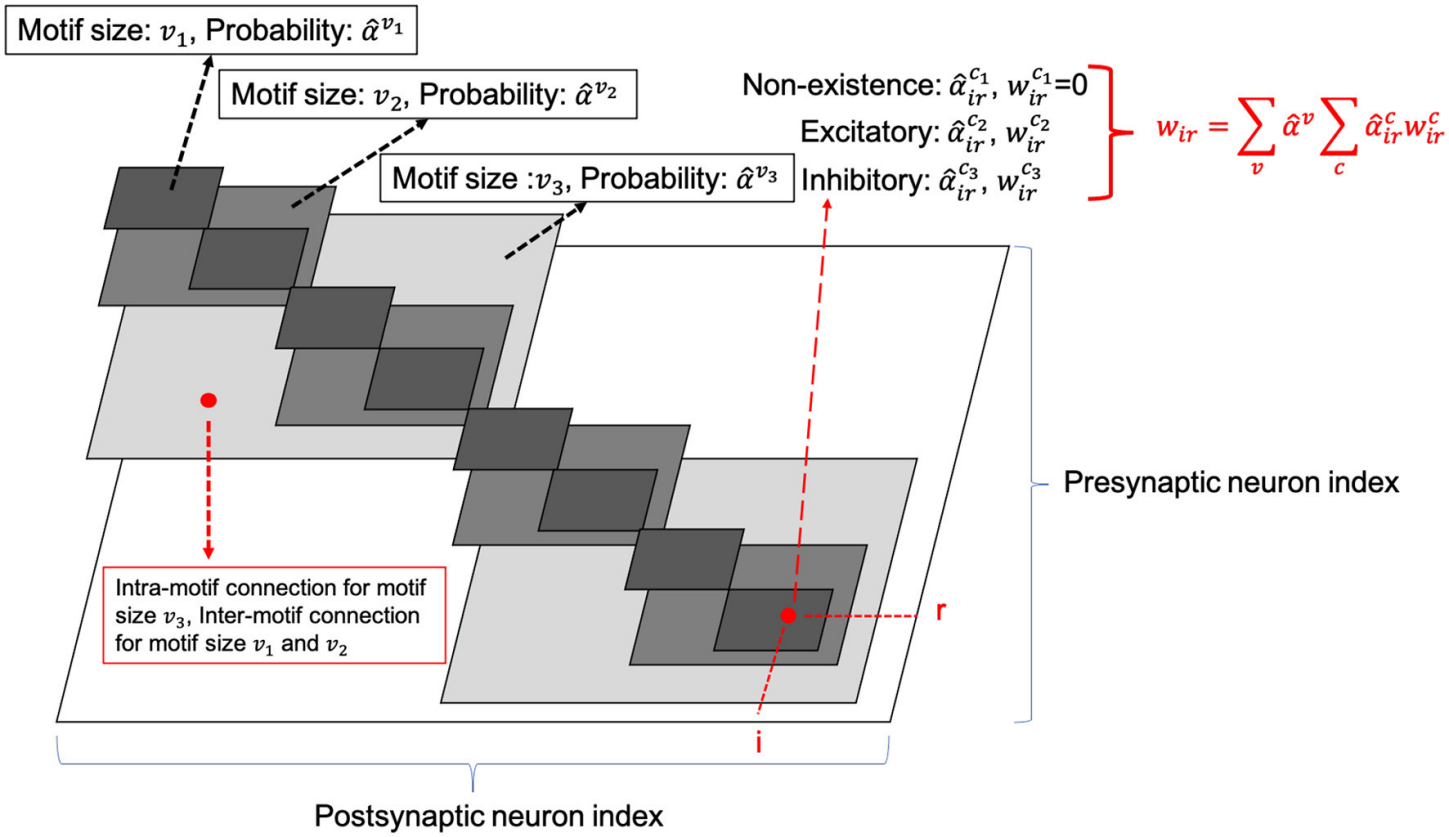
SC-ML with relaxed architectural parameters.

Optimizing the weight and architectural parameters by solving the bi-level optimization problem of Equations (5, 6, 7) can be computationally expensive. We adapt the recent method proposed in Liu et al. (2018) to reduce computational complexity by relaxing the discrete architectural parameters to continuous ones for efficient gradient-based optimization. Without loss of generality, we consider a multi-layered RSNN consisting of one or more SC-ML layers, where connections between layers are assumed to be feedforward. We focus on one SC-ML layer, as shown in Figure 5, to discuss the proposed gradient-based optimization.

The number of neurons in the SC-ML layer is fixed. The motif size is optimized such that each neuron is partitioned into a specific motif based on the chosen motif size. The largest white square in Figure 5 shows the layer-connectivity matrix of all intra-layer connections of the whole layer, where the dimension of the matrix corresponds to the neuron count of the layer. We superimpose three sets of smaller gray squares onto the layer-connectivity matrix, one for each of the three possible motif sizes of *v*_1_, *v*_2_, and *v*_3_ considered. Choosing a particular motif size packs neurons in the layer into multiple motifs, and the corresponding gray squares illustrate the intra-motif connectivity introduced within the SC-ML layer.

The entry of the layer-connectivity matrix at row *r* and column *i* specifies the existence and nature of the connection from neuron *r* to neuron *i*. We consider multiple motif size and connection type choices during architectural search using continuous-valued parameterizations α^*v*^ and $\alpha_{ir}^{c}$, respectively for each motif size *v* and connection type *c*. We relax the categorical choice of each motif size using a softmax over all possible options as ${\hat{\alpha }}^{v}$, and similarly relax the categorical choice of each connection type based on the corresponding motif size as ${\hat{\alpha }}_{ir}^{c}$:


(11) $$\begin{array}{l} {\hat{\alpha }}^{v}=\frac{exp\left(\alpha^{v}\right)}{\sum_{v^{\prime }\in V}exp\left(\alpha^{v^{\prime }}\right)},\;{\hat{\alpha }}_{ir}^{c}=\frac{exp\left(\alpha_{ir}^{c}\right)}{\sum_{c^{\prime }\in C}exp\left(\alpha_{ir}^{c^{\prime }}\right)} \end{array}$$

Here in Equation (11), V and C are the set of all motif sizes and possible connection types, respectively; ${\hat{\alpha }}^{v}$ and ${\hat{\alpha }}_{ir}^{c}$ are the continuous-valued categorical choice of motif size *v* and connection type *c*, respectively, which can also be interpreted as the probability of selecting the corresponding motif size or connection type. As in Figure 5, the synaptic weight of the connection from neuron *r* to neuron *i* is expressed as the summation of weights under all possible motif sizes and connection types weighted by the respective continuous-valued categorical choices (selection probabilities). In this paper, we use hat over the variable to denote the architectural parameter processed by softmax. Then, the task of architecture optimization is reduced to learn a set of continuous variables $\hat{\alpha }=\left\{{\hat{\alpha }}_{ir}^{c},{\hat{\alpha }}^{v}\right\}$. With the continuous architectural parameters, a gradient-based method like BP is applicable to learn the recurrent connectivity.

Since IP rules are independent of the network architecture search problem, in following derivation, we do not express the IP method parameters β explicitly and express the term $L_{valid}\left(\alpha ,w^{*}\left(\alpha \right),\beta_{-}^{*}\right)$ as $L_{valid}\left(\alpha ,w^{*}\left(\alpha \right)\right)$ for simplicity. In Liu et al. (2018), the bi-level optimization problem is simply approximated to a one-shot model to reduce the expensive computational cost of the inner optimization which can be expressed in Equation (12) as:


(12) $$\begin{array}{l} \nabla_{\hat{\alpha }}L_{valid}\left(\hat{\alpha },w^{*}\left(\hat{\alpha }\right)\right)=\nabla_{\hat{\alpha }}L_{valid}\left(\hat{\alpha },w-\eta \nabla_{w}L_{train}\left(w,\hat{\alpha }\right)\right), \end{array}$$

where η is the learning rate for a step of inner loop. Both the weights of the search network and the architectural parameters are trained by the BP method. The architectural gradient can be approximated in Equation (13):


(13) $$\begin{array}{l} \frac{d{ℒ}_{valid}}{d\hat{\alpha }}(\hat{\alpha })=\nabla_{\hat{\alpha }}{ℒ}_{valid}(\hat{\alpha },w^{*})-\eta \nabla_{w}{ℒ}_{valid}(\hat{\alpha },w^{*})\nabla_{\hat{\alpha },w}^{2}{ℒ}_{train}(w^{*},\hat{\alpha })). \end{array}$$

The complexity is further reduced by using the finite difference approximation around $w^{\pm }=w\pm \epsilon \nabla_{w}L_{valid}\left(\hat{\alpha },w^{*}\right)$ for small perturbation ϵ to compute the gradient of $\nabla_{\hat{\alpha }}L_{valid}\left(\hat{\alpha },w^{*}\right)$. Finally the architectural updates in (13) can be calculated as


(14) $$\begin{array}{l} \frac{d{ℒ}_{valid}}{d\hat{\alpha }}(\hat{\alpha })=\nabla_{\hat{\alpha }}{ℒ}_{valid}(\hat{\alpha },w^{*})-\frac{\eta }{2\epsilon }(\nabla_{\hat{\alpha }}{ℒ}_{train}(w^{+},\hat{\alpha })-\\ \;\nabla_{\hat{\alpha }}{ℒ}_{train}(w^{-},\hat{\alpha })). \end{array}$$

##### 2.3.3.2 Backpropagation via HRMAS framework

###### 2.3.3.2.1 Integrating architectural parameterizations into the LIF model

Based on the leaky integrate-and-fire (LIF) neuron model in Equation (3), the neuronal membrane voltage *u*_*i*_[*t*] of neuron *i* in the SC-ML layer at time *t* is given by integrating currents from all inter-layer inputs and intra-layer recurrent connections under all possible architectural parameterizations:


(15) $$\begin{array}{l} u_{i}^{-}[t]=(1-\frac{1}{\tau })u_{i}[t-1]+\frac{R}{\tau }(\sum_{j}w_{ij}a_{j}[t]+\\ \sum_{v\in V}({\hat{\alpha }}^{v}\sum_{r}^{I_{i}^{v}}\sum_{c\in C}\left({\hat{\alpha }}_{ir}^{c}w_{ir}^{c}a_{r}[t-1]))\right), \end{array}$$

where *R* and τ are the resistance and time constant of the membrane, *w*_*ij*_ the synaptic weight from neuron *j* in the previous layer to neuron *i*, $w_{ir}^{c}$ the recurrent weight from neuron *r* to neuron *i* of connection type *c*, and *a*_*j*_[*t*] the (unweighted) postsynaptic current (PSC) converted from spikes of neuron *j* through a synaptic model. To reduce clutter in the notation, we use $I_{i}^{v}$ to denote the number of presynaptic connections afferent onto neuron *i*'s input in the recurrent layer when choosing motif size *v*, which includes both inter and intra-motif connections and will be introduced in detail in the next paragraph. We further drop the explicit dependence of ${\hat{\alpha }}_{ir}^{c}$ on ${\hat{\alpha }}^{v}$. We assume feedforward connections have no time delay and recurrent connections have one time step delay. The response of neuron *i* obtained from recurrent connections is the summation of all the weighted recurrent inputs over the probabilities of connection types and motif sizes.

###### 2.3.3.2.2 SC-ML's topology and scalability

In this section we formally describe the topology of SC-ML and discuss its scalability. $I_{i}^{v}$ denote the number of presynaptic connections afferent onto neuron *i*'s input in the recurrent layer when choosing motif size *v*, and could be formally expressed as a union of inter-motif $I_{i,inter}^{v}$ and intra-motif $I_{i,intra}^{v}$ neuron connections (We have omitted the superscript of connection type c for convenience) in Equation (16):


(16) $$\begin{array}{l} I_{i}^{v}=I_{i,inter}^{v}\cup I_{i,intra}^{v} \end{array}$$

Hence the recurrent input weight of neuron *i* be expressed in Equation (17)


(17) $$\begin{array}{l} w_{ir}=w_{ir}^{inter}\cup w_{ir}^{intra} \end{array}$$

Let us consider a SC-ML layer with *N* neurons, divided into motif size = *v*, with *N*/*v* motifs within this layer. Let us denote the index of the motif by *k* ∈ (0, 1, 2, ..., *N*/*v* − 1). Assuming the neuron *i* is located in the *k*_*th*_ motif (i.e.: *kv* ≤ *i* ≤ *kv* + *v* − 1), then the intra-layer recurrent connection into neuron *i* be expressed as


$$w_{ir}^{intra},\text{where }r\in \left(kv,kv+1,kv+2,...,kv+v-1\right)$$

The inter-layer recurrent connection into neuron *i* be expressed as


$$w_{ir}^{inter},\text{where }r\in \left(i-v,i+v\right)$$

The figure expression is shown in Figure 1. The essence of SC-ML architecture design is to reduce the huge search space of the recurrent matrix and improve optimization efficiency through biologically inspired and carefully designed local recurrent connections as inductive bias. Hence, the SC-ML architecture can naturally adopt different inter and intra-motif topological connection patten across different layers, while providing scalability.

###### 2.3.3.2.3 Backpropagation in output layer

Through Equation (15), the continuous architecture parameterizations influence the integration of input currents, and hence firing activities of neurons in all layers and affect the loss function defined at the output layer. As such, the task of architecture optimization reduces to the one that learns the set of optimal continuous variables ${\hat{\alpha }}^{c}$ and ${\hat{\alpha }}^{v}$. The final architecture is constructed by choosing the parameterizations with the highest selection probabilities obtained from the optimization. During the learning, We define the loss function as in Equation (18)


(18) $$\begin{array}{l} L=\overset{T}{\underset{k=0}{\sum }}E\left[t_{k}\right], \end{array}$$

where *T* is the total time steps and *E*[*t*_*k*_] the loss at *t*_*k*_. From Equations (15) and (14), the membrane potential *u*_*i*_[*t*] of the neuron *i* at time *t* demonstrates contribution to all future fires and losses of the neuron through its PSC *a*_*i*_[*t*]. Therefore, the error gradient with respect to the presynaptic weight *w*_*ij*_ from neuron *j* to neuron *i* can be defined in Equation (19)


(19) $$\begin{array}{l} \begin{array}{l} \frac{\partial L}{\partial w_{ij}}=\overset{T}{\underset{k=0}{\sum }}\frac{\partial E\left[t_{k}\right]}{\partial w_{ij}}=\overset{T}{\underset{k=0}{\sum }}\overset{k}{\underset{m=0}{\sum }}\frac{\partial E\left[t_{k}\right]}{\partial u_{i}\left[t_{m}\right]}\frac{\partial u_{i}\left[t_{m}\right]}{\partial w_{ij}}\\ =\overset{T}{\underset{m=0}{\sum }}\frac{R}{\tau }a_{j}\left[t_{m}\right]\overset{T}{\underset{k=m}{\sum }}\frac{\partial E\left[t_{k}\right]}{\partial u_{i}\left[t_{m}\right]}=\overset{T}{\underset{m=0}{\sum }}\frac{R}{\tau }a_{j}\left[t_{m}\right]\delta_{i}\left[t_{m}\right], \end{array} \end{array}$$

where δ_*i*_[*t*_*m*_] denotes the error for neuron *i* at time *t*_*m*_ and is defined in Equation (20):


(20) $$\begin{array}{l} \delta_{i}\left[t_{m}\right]=\overset{T}{\underset{k=m}{\sum }}\frac{\partial E\left[t_{k}\right]}{\partial u_{i}\left[t_{m}\right]}=\overset{T}{\underset{k=m}{\sum }}\frac{\partial E\left[t_{k}\right]}{\partial a_{i}\left[t_{k}\right]}\frac{\partial a_{i}\left[t_{k}\right]}{\partial u_{i}\left[t_{m}\right]}. \end{array}$$

In this work, the output layer is regular feedforward layer without recurrent connection. Therefore, the weight *w*_*oj*_ of output neuron *o* is updated by Equation (21)


(21) $$\begin{array}{l} \frac{\partial L}{\partial w_{oj}}=\overset{T}{\underset{m=0}{\sum }}\frac{R}{\tau }a_{j}\left[t_{m}\right]\overset{T}{\underset{k=m}{\sum }}\frac{\partial E\left[t_{k}\right]}{\partial a_{o}\left[t_{k}\right]}\frac{\partial a_{o}\left[t_{k}\right]}{\partial u_{o}\left[t_{m}\right]}, \end{array}$$

where $\frac{\partial E\left[t_{k}\right]}{\partial a_{o}\left[t_{k}\right]}$ depends on the choice of the loss function.

###### 2.3.3.2.4 Backpropagation in hidden layers

Now, we focus on the backpropagation in the recurrent hidden layer while the feedforward hidden layer case can be derived similarly. For a neuron *i* in SC-ML, in addition to the error signals from the next layer, the error backpropagated from the recurrent connections should also be taken into consideration. The backpropagated error can be calculated by:


(22) $$\begin{array}{l} \begin{array}{l} \delta_{i}\left[t_{m}\right]=\overset{T}{\underset{k=m}{\sum }}\overset{T}{\underset{j=k}{\sum }}\frac{\partial a_{i}\left[t_{k}\right]}{\partial u_{i}\left[t_{m}\right]}\overset{N_{p}}{\underset{p=1}{\sum }}\left(\frac{\partial u_{p}\left[t_{k}\right]}{\partial a_{i}\left[t_{k}\right]}\frac{\partial E\left[t_{j}\right]}{\partial u_{p}\left[t_{k}\right]}\right)+\\ \overset{T}{\underset{k=m}{\sum }}\overset{T}{\underset{j=k+1}{\sum }}\frac{\partial a_{i}\left[t_{k}\right]}{\partial u_{i}\left[t_{m}\right]}\overset{N_{r}}{\underset{r}{\sum }}\left(\frac{\partial u_{r}\left[t_{k}+1\right]}{\partial a_{i}\left[t_{k}\right]}\frac{\partial E\left[t_{j}\right]}{\partial u_{r}\left[t_{k}+1\right]}\right)\\ =\overset{T}{\underset{k=m}{\sum }}\frac{\partial a_{i}\left[t_{k}\right]}{\partial u_{i}\left[t_{m}\right]}\overset{N}{\underset{p=1}{\sum }}\left(\frac{R}{\tau }w_{pi}\delta_{p}\left[t_{k}\right]\right)+\\ \overset{T-1}{\underset{k=m}{\sum }}\frac{\partial a_{i}^{\left(l\right)}\left[t_{k}\right]}{\partial u_{i}^{\left(l\right)}\left[t_{m}\right]}\underset{v\in V}{\sum }\left({\hat{\alpha }}^{v}\overset{O_{i}^{v}}{\underset{r}{\sum }}\underset{c\in C}{\sum }\frac{R}{\tau }{\hat{\alpha }}_{ri}^{c}w_{ri}^{c}\delta_{r}\left[t_{k}+1\right]\right), \end{array} \end{array}$$

where *N*_*p*_ and *N*_*r*_ are the number of neurons in the next layer and the number of neurons in this recurrent layer, respectively. δ_*p*_ and δ_*r*_ are the errors of the neuron *p* in the next layer and the error from the neuron *r* through the recurrent connection. $O_{i}^{v}$ represents all the postsynaptic neurons of neuron *i*'s outputs in the recurrent layer when choosing motif size *v*, which includes both inter and intra-motif connections.

The key term in Equation (22) is $\frac{\partial a\left[t_{k}\right]}{\partial u\left[t_{m}\right]}$ which reflects the effect of neuron's membrane potential on its output PSC. Due to the non-differentiable spiking events, it becomes the main difficulty for the BP of SNNs. Various approaches are proposed to handle this problem such as probability density function of spike state change (Shrestha and Orchard, 2018), surrogate gradient (Neftci et al., 2019), and Temporal Spike Sequence Learning via Backpropagation (TSSL-BP) (Zhang and Li, 2020). In our experiments, we adopt the TSSL-BP method to calculated $\frac{\partial a\left[t_{k}\right]}{\partial u\left[t_{m}\right]}$. With the error backpropagated according to Equation (22), the weights and architectural parameters can be updated by gradient descent as:


(23) $$\begin{array}{l} \begin{array}{l} \Delta w_{ij}\propto \delta_{i}\left[t\right]\frac{R}{\tau }a_{j}\left[t\right],\;\Delta {\hat{\alpha }}^{v}\propto \overset{N_{r}}{\underset{i}{\sum }}\delta_{i}\left[t\right]\frac{R}{\tau }\overset{I_{i}^{v}}{\underset{r}{\sum }}\left(\underset{c\in C}{\sum }{\hat{\alpha }}_{ir}^{c}w_{ir}^{c}a_{r}\left[t-1\right]\right),\\ \Delta w_{ir}^{c}\propto \delta_{i}\left[t\right]\frac{R}{\tau }\underset{v\in V}{\sum }\left({\hat{\alpha }}^{v}{\hat{\alpha }}_{ir}^{c}a_{r}\left[t-1\right]\right),\\ \;\Delta {\hat{\alpha }}_{ir}^{c}\propto \delta_{i}\left[t\right]\frac{R}{\tau }\underset{v\in V}{\sum }\left({\hat{\alpha }}^{v}w_{ir}^{c}a_{r}\left[t-1\right]\right). \end{array} \end{array}$$

where δ_*i*_[*t*] is the backpropagated error for neuron *i* at time *t* given in Equation (22), *N*_*r*_ is the number of neurons in this recurrent layer, *R* and τ are the leaky resistance and membrane time constant, two intrinsic parameters adapted by the IP rule, *a*_*j*_[*t*] and *a*_*r*_[*t*] are the (unweighted) postsynaptic currents (PSCs) generated based on synpatic model by the presynaptic neuron *j* in the preceding layer and the *r*-th neuron in this recurrent layer, respectively.

#### 2.3.4 Risk minimizing optimization with intrinsic plasticity

For architectural optimization of non-spiking RNNs, gradient-based methods are shown to be unstable in some cases due to misguided architectural changes and conversion from the optimized continuous-valued parameterization to a discrete architectural solution, hindering the final performance and demolishing the effectiveness of learning (Zela et al., 2019). Adaptive regularization which modifies the regularization strength (weight decay) guided by the largest eigenvalue of $\nabla_{\alpha }^{2}L_{valid}$ was proposed to address this problem (Zela et al., 2019). While this method shows promise for non-spiking RNNs, it is computationally intensive due to frequent expensive eigenvalue computation, severely limiting its scalability.

To address risks observed in architectural changes for RSNNs, we introduce a biologically-inspired risk-mitigation method. Biological circuits demonstrate that Intrinsic Plasticity (IP) is crucial in reducing such risks. IP is a self-regulating mechanism in biological neurons ensuring homeostasis and influencing neural circuit dynamics (Marder et al., 1996; Baddeley et al., 1997; Desai et al., 1999). IP is based on local neural firing activities and performs online adaptation with minimal additional computational overhead. It not only stabilizes neuronal activity but also coordinates connectivity and excitability changes across neurons to stabilize circuits (Maffei and Fontanini, 2009; Tien and Kerschensteiner, 2018). IP has been applied in spiking neural networks for locally regulating neuron activity (Lazar et al., 2007; Bellec et al., 2018). In Zhang et al. (2019), the application of IP mechanism significantly improves computational performance in terms of learning speed, accuracy, and robustness to input variations and noise. Fourati et al. (2020) proposes a deep echo state network that utilizes intrinsic plasticity to drive reservoir neuron activities to follow a desired Gaussian distribution, enabling the learning of discriminative EEG representations and demonstrating its effectiveness on emotion recognition benchmarks. Zhang et al. (2020) proposes a novel IP learning rule based on a soft-reset spiking neuron model, which ensures the neuron's membrane potential is mathematically continuous and differentiable. Experimental results demonstrate that the proposed IP rule can effectively improve the classification accuracy, inference speed, and noise robustness. Zhang et al. (2021) proposes input-driven and self-driven intrinsic IP learning rules for spiking convolutional neural networks (SCNNs), where IP updates occur only when a neuron receives input spikes or generates an output spike, respectively. Experiments show that the event-driven IP rules significantly reduce IP update operations and accelerate convergence while maintaining accuracy.

Drawing from these findings, we make use of IP for mitigating the risk of RSNN architectural modifications in this work. Our HRMAS framework integrates the IP rule into the architectural optimization, applied in the second step of each iteration. We adopt the SpiKL-IP rule (Zhang and Li, 2019a) for all recurrent neurons during architecture optimization. SpiKL-IP adapts the intrinsic parameters of a spiking neuron while minimizing the KL-divergence from the output firing rate distribution to a targeted exponential distribution. It both maintains a level of network activity and maximizes the information transfer for each neuron. We adapt leaky resistance and membrane time constant of each neuron using SpiKL-IP which effectively solves the optimization problem in Equation (6) in an online manner as Equation (24):


(24) $$\begin{array}{l} \Delta R=\frac{2y\tau V_{th}-W-V_{th}-\frac{1}{\mu }\tau V_{th}y^{2}}{RW},\;\\ \Delta \tau =\frac{-1+\frac{y}{\mu }}{\tau },\;W=\frac{V_{th}}{e^{\frac{1}{\tau y}}-1}, \end{array}$$

where μ is the desired mean firing rate, *y* the average firing rate of the neuron. Similar to biological neurons, we use the intracellular calcium concentration ϕ[*t*] as a good indicator of the averaged firing activity and y can be expressed with the time constant of calcium concentration τ_*cal*_ as Equation (25)


(25) $$\begin{array}{l} \phi_{i}\left[t\right]=\left(1-\frac{1}{\tau_{cal}}\right)\phi_{i}\left[t-1\right]+s_{i}\left[t\right],\;y_{i}\left[t\right]=\frac{\phi_{i}\left[t\right]}{\tau_{cal}}. \end{array}$$

We explicitly express the neuronal parameters *R* and τ of neuron *i* tuned through time as *R*_*i*_[*t*] and τ_*i*_[*t*], since they are adjusted by the IP rule at each time step. They are updated by Equation (26)


(26) $$\begin{array}{l} R_{i}\left[t\right]=R_{i}\left[t-1\right]-\gamma \Delta R_{i},\;\tau_{i}\left[t\right]=\tau_{i}\left[t-1\right]-\gamma \Delta \tau_{i}, \end{array}$$

where γ is the learning rate of the SpiKL-IP rule. By including time-variant neuronal parameters *R* and τ into Equations (22) and (23), the one time step architectural parameter and weight updated by Equations (27, 28)


(27) $$\begin{array}{l} \begin{array}{l} \delta_{i}\left[t_{m}\right]=\overset{T}{\underset{k=m}{\sum }}\frac{\partial a_{i}\left[t_{k}\right]}{\partial u_{i}\left[t_{m}\right]}\overset{N}{\underset{p=1}{\sum }}\left(\frac{R_{p}\left[t_{k}\right]}{\tau_{p}\left[t_{k}\right]}w_{pi}\delta_{p}\left[t_{k}\right]\right)\\ +\overset{T-1}{\underset{k=m}{\sum }}\frac{\partial a_{i}^{\left(l\right)}\left[t_{k}\right]}{\partial u_{i}^{\left(l\right)}\left[t_{m}\right]}\underset{v\in V}{\sum }\left({\hat{\alpha }}^{v}\overset{O_{i}^{v}}{\underset{r}{\sum }}\underset{c\in C}{\sum }\frac{R_{r}\left[t_{k}+1\right]}{\tau_{r}\left[t_{k}+1\right]}{\hat{\alpha }}_{ri}^{c}w_{ri}^{c}\delta_{r}\left[t_{k}+1\right]\right) \end{array} \end{array}$$

(28) $$\begin{array}{l} \begin{array}{l} \Delta w_{ij}\propto \delta_{i}\left[t\right]\frac{R_{i}\left[t\right]}{\tau_{i}\left[t\right]}a_{j}\left[t\right],\;\\ \Delta {\hat{\alpha }}^{v}\propto \overset{N_{r}}{\underset{i}{\sum }}\delta_{i}\left[t\right]\frac{R_{i}\left[t\right]}{\tau_{i}\left[t\right]}\overset{I_{i}^{v}}{\underset{r}{\sum }}\left(\underset{c\in C}{\sum }{\hat{\alpha }}_{ir}^{c}w_{ir}^{c}a_{r}\left[t-1\right]\right),\\ \Delta w_{ir}^{c}\propto \delta_{i}\left[t\right]\frac{R_{i}\left[t\right]}{\tau_{i}\left[t\right]}\underset{v\in V}{\sum }\left({\hat{\alpha }}^{v}{\hat{\alpha }}_{ir}^{c}a_{r}\left[t-1\right]\right),\;\\ \Delta {\hat{\alpha }}_{ir}\propto \delta_{i}\left[t\right]\frac{R_{i}\left[t\right]}{\tau_{i}\left[t\right]}\underset{v\in V}{\sum }\left({\hat{\alpha }}^{v}w_{ir}^{c}a_{r}\left[t-1\right]\right), \end{array} \end{array}$$

The proposed alternating two-step optimization of HRMAS is summarized in Algorithm 1. Architectural parameters α includes size of motif, and type of motif connections. They are optimized separately in two consecutive stages. We express here only a formal unification, for the sake of clarity in the architecture search problem.

**Algorithm 1:** Hybrid risk-mitigating architectural search.

Initialize weights *w*, intrinsic parameters β, architectural parameters α, and correspondingly $\hat{\alpha }$.
**repeat**
Update $\hat{\alpha }$ by
$\eta_{1}\nabla_{\hat{\alpha }}L_{valid}\left(\hat{\alpha },w-\eta_{2}\nabla_{w}L_{train}\left(\hat{\alpha },w,\beta \right)\right)$;
Update *w* by $\eta_{2}\nabla_{w}L_{train}\left(\hat{\alpha },w,\beta \right)$;
$\beta \leftarrow \text{SpiKL-IP}\left(\hat{\alpha },w\right)$;
**until** converged

## 3 Results

The proposed HRMAS optimized RSNNs with the SC-ML layer architecture and five motif size options are evaluated on speech dataset TI46-Alpha (Liberman et al., 1991), neuromorphic speech dataset N-TIDIGITS (Anumula et al., 2018), neuromorphic video dataset DVS-Gesture (Amir et al., 2017), and neuromorphic image dataset N-MNIST (Orchard et al., 2015). The performances are compared with recently reported state-of-the-art manually designed architectures of SNNs and ANNs such as feedforward SNNs, RSNNs, LSM, and LSTM. For the proposed work, the architectural parameters are optimized by HRMAS with the weights trained on a training set and architectural parameters learned on a validation set as shown in Algorithm 1. The accuracy of each HRMAS optimized network is evaluated on a separate testing set with all weights reinitialized. **Table 2** shows all results.

### 3.1 Experimental settings

#### 3.1.1 Dataset

The proposed HRMAS framework with SC-ML is evaluated on speech dataset TI46-Alpha (Liberman et al., 1991), neuromorphic speech dataset N-TIDIGITS (Anumula et al., 2018), neuromorphic video dataset DVS-Gesture (Amir et al., 2017), and neuromorphic image dataset N-MNIST (Orchard et al., 2015). The performances are compared with several existing results on different structures of SNNs and ANNs such as feedforward SNNs, RSNNs, Liquid State Machine(LSM), LSTM, and so on.

#### 3.1.2 Loss function

For the BP method used in this work, the loss function can be defined by any errors that measure the distance between the actual outputs and the desired outputs. In our experiments, since hundreds of time steps are required for simulating speech and neuromorphic inputs, we choose the accumulated output PSCs to define the error which is similar to the firing count used in many existing works (Jin et al., 2018; Shrestha and Orchard, 2018). We suppose the simulation time steps for a sample is *T*. In addition, for neuron *o* of the output layer, we define the desired output as *d*_*o*_ = (*d*_*o*_[*t*_0_], *d*_*o*_[*t*_1_]...., *d*_*o*_[*t*_*N*_]) and real output as *a*_*o*_ = (*a*_*o*_[*t*_0_], *a*_*o*_[*t*_1_]...., *a*_*o*_[*t*_*N*_]) and *d*_*o*_ is manually determined. Therefore, the loss is determined by the square error of the outputs


(29) $$\begin{array}{l} L=\overset{T}{\underset{k=1}{\sum }}E\left[t_{k}\right]=\overset{T}{\underset{k=1}{\sum }}\overset{N^{\left(out\right)}}{\underset{o}{\sum }}\frac{1}{2}{\left(d_{o}\left[t_{k}\right]-a_{o}\left[t_{k}\right]\right)}^{2} \end{array}$$

where *N*^(*out*)^ is the number of neurons in the output layer and *E*[*t*_*k*_] is the error at time step *t*_*k*_, which is simply defined by the averaged loss through all the time steps in Equation (30)


(30) $$\begin{array}{l} E\left[t_{k}\right]≜\overset{N^{\left(out\right)}}{\underset{o}{\sum }}\frac{1}{2}{\left(d_{o}\left[t_{k}\right]-a_{o}\left[t_{k}\right]\right)}^{2}. \end{array}$$

With the loss function defined above, the error δ can be calculated for each layer according to Equation (22). We use a manually specified target output sequence to calculate the loss. Typically, we want neurons in a target class to fire at every timestep with spike train output: (1,1, ....,1), while neurons in other classes are silenced with spike train output: (0,0, ....,0). Loss is then calculated by comparing the target spike train's PSC *d*_*o*_ with the actual spike train's PSC *a*_*o*_ in Equation (29).

#### 3.1.3 Network architecture and hyperparameters

In the SNNs of the experiments, the fully connected weights between layers are initialized by the He Normal initialization proposed in He et al. (2015). The recurrent weights of excitatory connections are initialized to 0.2 and tuned by the BP method. The weights of inhibitory connections are initialized to −2 and fixed. The simulation step size is set to 1 ms. The parameters like thresholds and learning rate are empirically tuned. No synaptic delay is applied for feedforward connections while recurrent connections have 1 time step delay. No refractory period, normalization, or dropout is used. Kingma and Ba (2014) is adopted as the optimizer. The mean and standard deviation (std) of the accuracy reported is obtained by repeating the experiments five times.

Table 1 lists the typical constant values of parameters adopted in our experiments for each dataset. The SC-ML size denotes the number of neurons in the SC-ML. In our experiments, each network contains one SC-ML as the hidden layer. In addition, five motif sizes are predetermined before the experiment. The HRMAS framework optimizes the motif size from one of the five options.

**Table 1:** Parameters settings.

**Parameter**	**TI46-Alpha**	**N-TIDIGITS**	**DvsGesture**	**N-MNIST**
τ_*m*_	16 ms	64 ms	64 ms	16 ms
τ_*s*_	8 ms	8 ms	8 ms	8 ms
τ_*cal*_	16 ms	16 ms	16 ms	16 ms
Learning rate	0.0005	0.0005	0.0001	0.0005
Batch size	50	50	20	50
Time steps	100	300	400	100
Epochs for searching	300	200	60	30
Epochs for testing	400	400	150	100
SC-ML size	800	800	512	512
Motif size options	[5, 10, 16, 25, 40]		[2, 4, 8, 16, 32]	

Bold indicates the highest accuracy.

#### 3.1.4 Traing process

Our experiments contain two phases. In the first phase, the weights are trained via the training set while the validation set is used to optimize architectural parameters. In the second phase, the motif topology and type of lateral connections are fixed after obtaining the optimal architecture. All the weights of the network are reinitialized. Then, the new network is trained on the training set and tested on the testing set. The test performance is reported in the paper. In addition, since all the datasets adopted in this paper only contain training sets and testing sets, our strategy is to divide the training set. In the first phase, the training set is equally divided into a training subset and a validation subset. Then, the architecture is optimized on these subsets. In the second phase, since all the weights are reinitialized, we can train the weights with the full training set and test on the testing set. Note that the testing set is only used for the final evaluation.

### 3.2 Performance of HRMAS

Table 2 shows the results on the TI46-Alpha dataset. In order to verify the performance of the HRMAS algorithm, we conducted five experiments on each dataset (using different initialization seeds) and recorded the highest accuracy, average accuracy and standard deviation. The HRMAS-optimized RSNN has one hidden SC-ML layer with 800 neurons, and outperforms all other models while achieving 96.44% accuracy with mean of 96.08% and standard deviation (std) of 0.27% on the testing set. The proposed RSNN outperforms the LSM model in Wijesinghe et al. (2019) by 18.44%. It also outperforms the larger multi-layered RSNN with more tunable parameters in Zhang and Li (2019b) trained by the spike-train level BP (ST-RSBP) by 3.1%. Recently, Zhang and Li (2021b) demonstrated improved performances from manually designed RNNs with self-recurrent connections trained using the same TSSL-BP method. Our automated HRMAS architectural search also produces better performing networks.

**Table 2:** Accuracy on TI46-Alpha, N-TIDIGITS, DVS-Gesture and N-MNIST.

**Dataset**	**Network structure**	**Learning rule**	**Hidden layers**	**Best**
TI46-Alpha	LSM (Wijesinghe et al., 2019)	Non-spiking BP	2,000	78%
	RSNN (Zhang and Li, 2019b)	ST-RSBP	400 − 400 − 400	93.35%
	Sr-SNN (Zhang and Li, 2021b)	TSSL-BP	400 − 400 − 400	94.62%
	This work	TSSL-BP	800	**96.44%**
N-TIDIGITS	GRU (Anumula et al., 2018)	Non-spiking BP	200 − 200 − 100	90.90%
	Phase LSTM (Anumula et al., 2018)	Non-spiking BP	250 − 250	91.25%
	RSNN (Zhang and Li, 2019b)	ST-RSBP	400 − 400 − 400	93.90%
	Feedforward SNN	TSSL-BP	400	84.84%
	This work	TSSL-BP	400	**94.66%**
DVS-Gesture	Feedforward SNN (He et al., 2020)	STBP	*P*4 − 512	87.50%
	LSTM (He et al., 2020)	Non-spiking BP	*P*4 − 512	88.19%
	HeNHeS	STDP	500	90.15%
	Feedforward SNN	TSSL-BP	*P*4 − 512	88.19%
	This work	TSSL-BP	*P*4 − 512	**90.28**%
N-MNIST	Feedforward SNN (He et al., 2020)	STBP	512	98.19%
	RNN (He et al., 2020)	Non-spiking BP	512	98.15%
	LSTM (He et al., 2020)	Non-spiking BP	512	98.69%
	ELSM(Pan et al., 2024)	Non-spiking BP	8000	97.23%
	This work	TSSL-BP	512	**98.72**%

HeNHeS result is from Chakraborty and Mukhopadhyay (2023). Bold indicates the highest accuracy.

We also show that a HRMAS-optimized RSNN with a 400-neuron SC-ML layer outperforms several state-of-the-art results on the N-TIDIGITS dataset (Zhang and Li, 2019b), achieving 94.66% testing accuracy (mean: 94.27%, std: 0.35%). Our RSNN has more than a 3% performance gain over the widely adopted recurrent structures of ANNs, the GRU and LSTM. It also significantly outperforms a feedforward SNN with the same hyperparameters, achieving an accuracy improvement of almost 9.82%, demonstrating the potential of automated architectural optimization.

**Figure 6:**
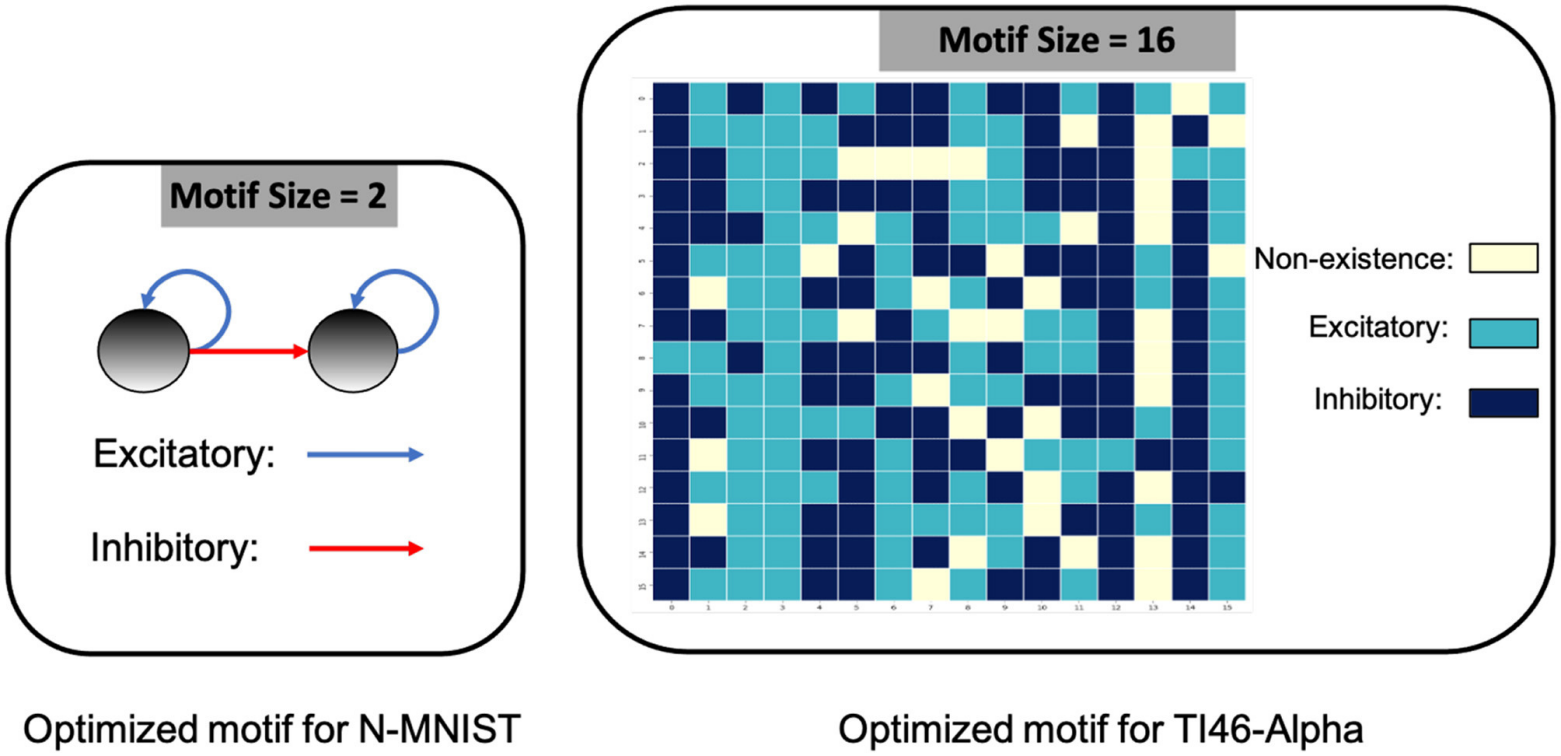
Optimized motif topologies.

**Figure 7:**
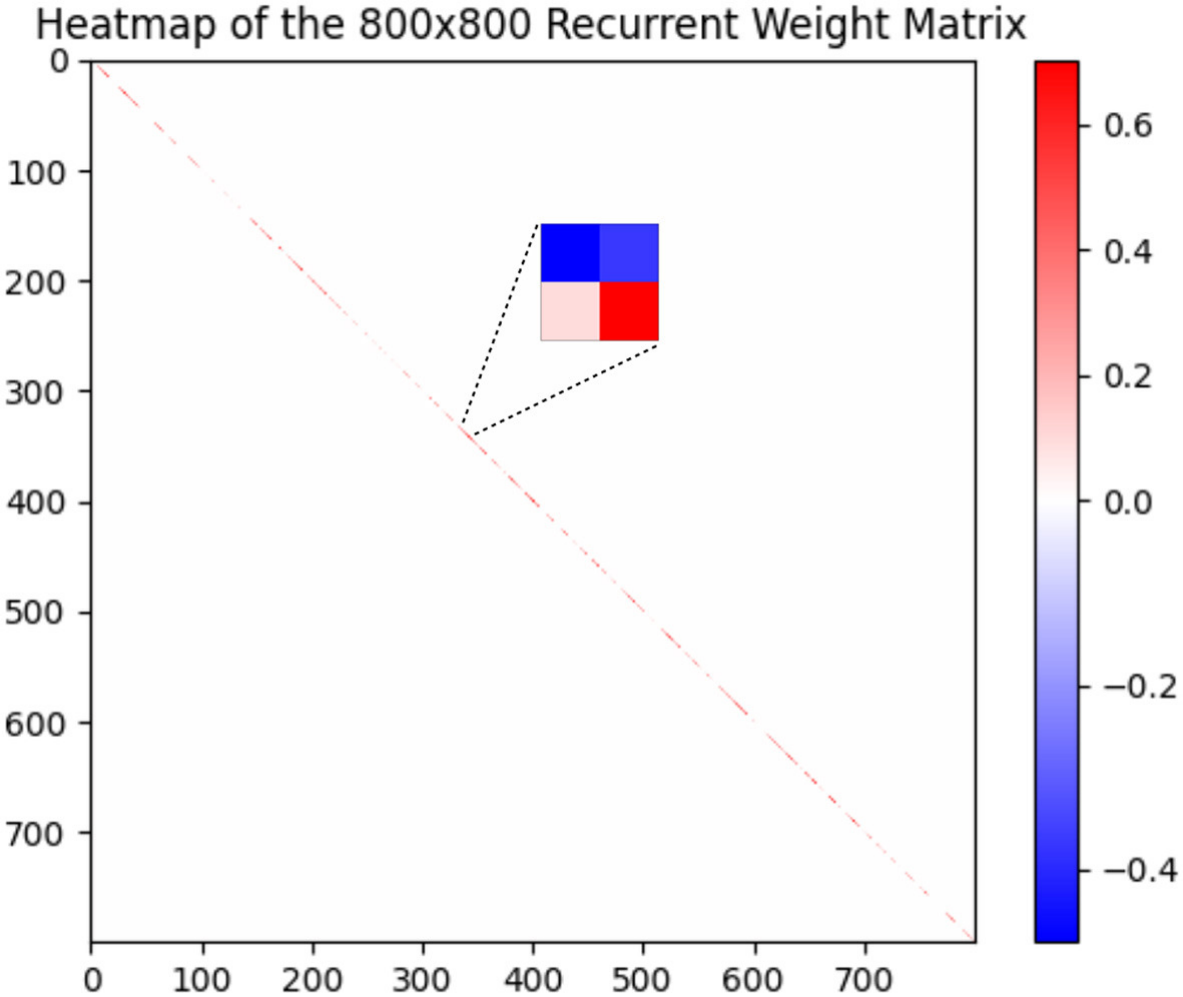
Recurrent Weight Matrix after optimization by HRMAS.

On DVS-Gesture and N-MNIST, our method achieves accuracies of 90.28% (mean: 88.40%, std: 1.71%) and 98.72% (mean: 98.60%, std: 0.08%), respectively. Table 2 compares a HRMAS-optimized RSNN with models including feedforward SNNs trained by TSSL-BP (Zhang and Li, 2020) or STBP (Wu et al., 2018) with the same size, and non-spiking ANNs vanilla LSTM (He et al., 2020). Note that although our RSNN and the LSTM model have the same number of units in the recurrent layer, the LSTM model has a much greater number of tunable parameters and a improved rate-coding-inspired loss function. Our HRMAS-optimized model surpasses all other models. For a more intuitive understanding, Figure 6 presents two examples of the motif topology optimized by HRMAS: motif sizes 2 in options [2, 4, 8, 16, 32] for the N-MNIST dataset and motif size 16 in options [5, 10, 16, 25, 40] for the TI-Alpha dataset. We also shows in the Figure 7 the weight matrix of the RSNN with SC-ML optimized by the HRMAS method. The original fully connected recurrent matrix size is 800*800. We set the search space of motif size to [2,4,8,16,32]. In five random experiments, the HRMAS optimization method always gave the search results of motif-size = 2, with similar inter/intra motif topology. This limits the huge recurrent matrix to a highly sparse band matrix with non-zero values only near the diagonal, greatly reducing the search space, parameter amount, and optimization difficulty.

### 3.3 Ablation analysis

#### 3.3.1 Ablation experiments of proposed components

We conduct ablation studies on the RSNN optimized by HRMAS for the TI46-Alpha dataset to reveal the contributions of various proposed techniques. When all proposed techniques are included, the HRMAS-optimized RSNN achieves 96.44% accuracy. In Table 3, removing of the IP rule from the second step of the HRMAS optimization iteration visibly degrades the performance, showing the efficacy of intrinsic plasticity for mitigating risks of architectural changes. A similar performance degradation is observed when the sparse inter-motif connections are excluded from the SC-ML layer architecture. Without imposing a structure in the hidden layer by using motifs as a basic building block, HRMAS can optimize all possible connectivity types of the large set of 800 hidden neurons. However, this creates a large and highly complex architectural search space, rendering a tremendous performance drop. Finally, we compare the HRMAS model with an RSNN of a fixed architecture with full recurrent connectivity in the hidden layer. The application of the BP method is able to train the latter model since no architectural (motifs or connection types) optimization is involved. However, albeit its significantly increased model complexity due to dense connections, this model has a large performance drop in comparison with the RSNN fully optimized by HRMAS. We provide additional data including ablation experiments, the computational resources required by our method, and the IP rule's effect on performance during optimizing process in Section 3.3.1.

**Table 3:** Ablation studies of HRMAS on TI46-Alpha.

**Setting**	**Accuracy**
Full HRMAS	**96.44%**
Without IP	95.20%
Without motif	88.35%
Without inter-motif connections	95.73%
Fully connected RSNN	94.10%

Bold indicates the highest accuracy.

#### 3.3.2 Ablation experiments of network parameters

We provided additional ablation experiments in Table 4, including: random search in the search space as the baseline for HRMAS, effect of IP rule, HRMAS perfermence on larger network. Experimental results show that: the HRMAS method shows consistent superiority (around 2%) over the random search baseline; IP rule brings stable performance improvement (around 1.3%); our method can be efficiently extended to networks with more neurons while providing good performance.

**Table 4:** Test Accuracy on TI46-Alpha, obtained by repeating five times with different random seeds, including: HRMAS perfermence on Larger network, effect of IP rule, random search as the baseline architecture.

**Arch optimization**	**Learning rule**	**SC-ML sizes**	**Best**	**Mean**	**Std**
HRMAS (with IP)	TSSL-BP	800	96.44%	96.08%	0.27%
HRMAS (with IP)	TSSL-BP	1, 600	96.26%	–	–
HRMAS (with IP)	TSSL-BP	2, 400	**96.58%**	–	–
HRMAS (with IP)	TSSL-BP	3, 200	96.45%	–	–
HRMAS (w/o IP)	TSSL-BP	800	95.17%	94.74%	0.32%
Random	TSSL-BP	800	94.47%	94.18%	0.30%

Bold indicates the highest accuracy.

### 3.4 IP rule's effect on performance during optimizing process

**Figure 8:**
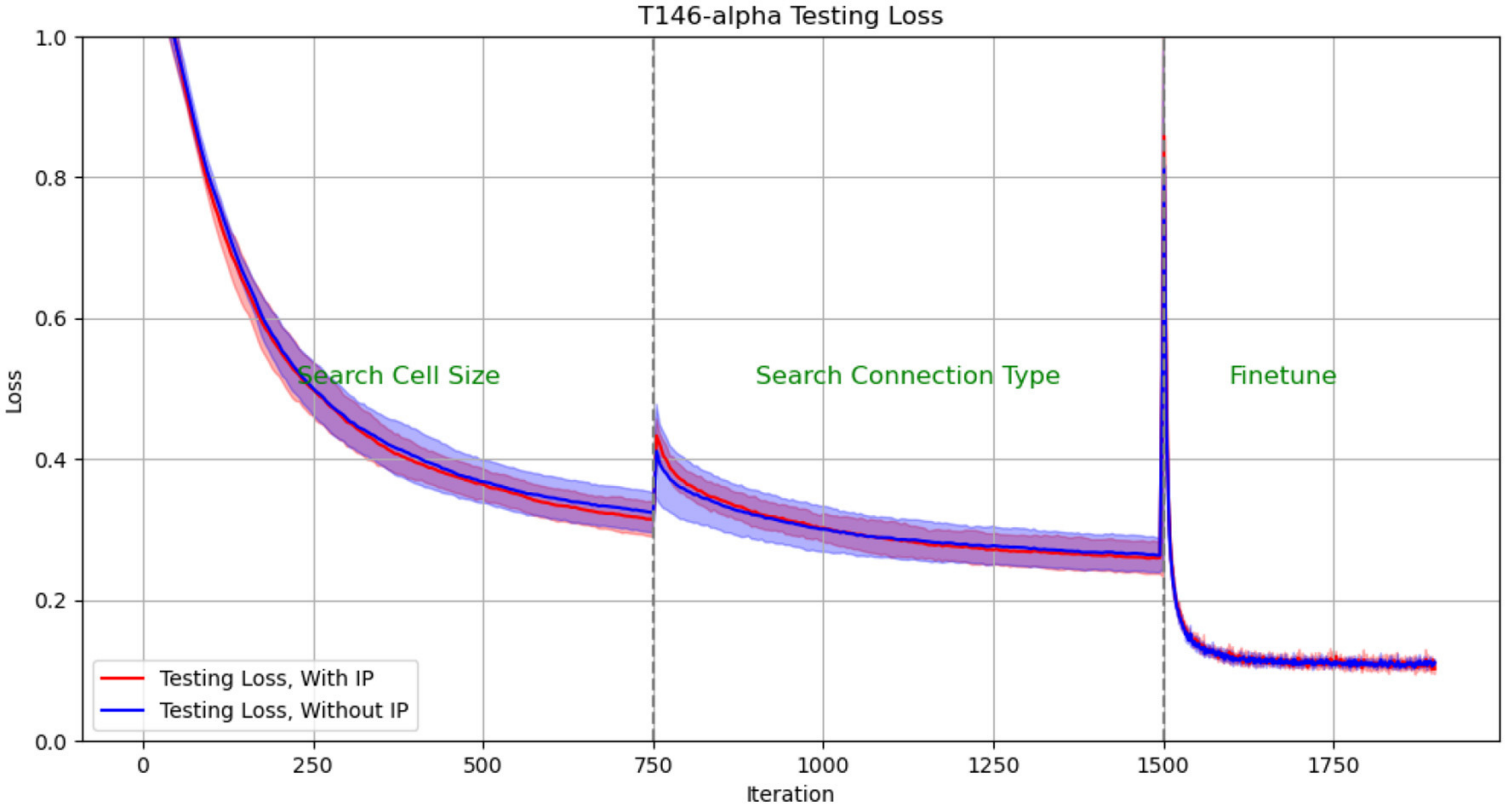
Test Loss in architectural optimization in HRMAS. The solid line and shading show the mean and standard deviation of the 5 experiments. We conducted experiments with ip rule turned on (red) and off (blue). It can be found that IP method brings two benefits: improved network performance and a more stable training process. The figures showed that the red solid line (mean) has lower loss than the blue solid line without the IP method; at the same time, the red shadow (standard deviation) has always been narrower than the blue shadow, which means a more stable network architecture search process.

**Figure 9:**
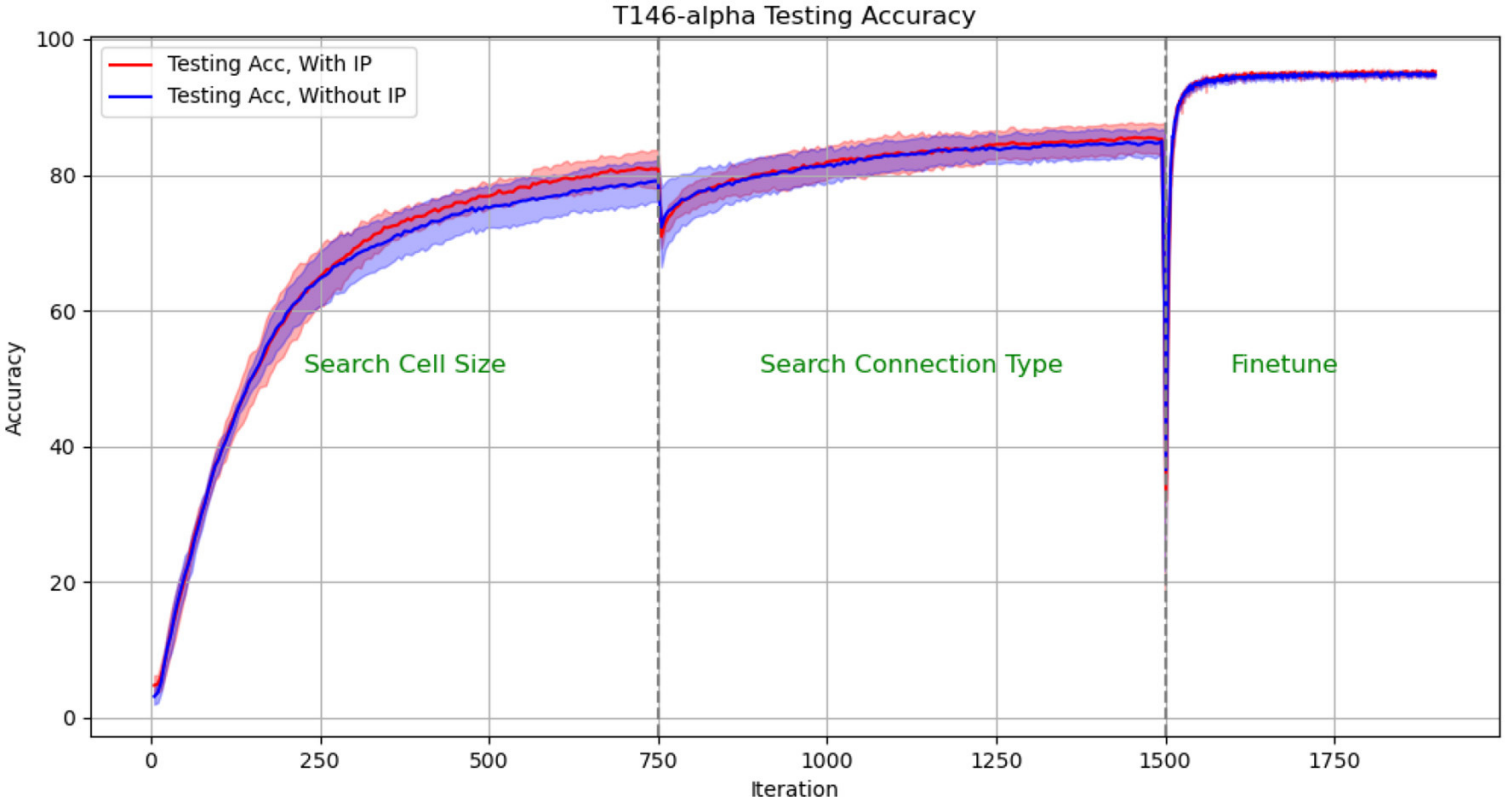
Test Accuracy in architectural optimization in HRMAS. The solid line and shading show the mean and standard deviation of the 5 experiments. We conducted experiments with ip rule turned on (red) and off (blue). It can be found that IP method brings two benefits: improved network performance and a more stable training process. The figures showed that the red solid line (mean) has higher accuracy than the blue solid line without the IP method; at the same time, the red shadow (standard deviation) has always been narrower than the blue shadow, which means a more stable network architecture search process.

We plotted the performance curve of the network optimization process on the TI46-alpha dataset. Figures 8, 9 show the loss and accuracy on the validation set respectively. The solid line and shading show the mean and standard deviation of the five experiments. We conducted experiments with IP rule turned on and off. We use green text to mark each phase of architecture optimization.

The experimental results show: (1) When the network architecture changes drastically, such as iteration = 750 (the cell size search ends and the connection type search starts), and iteration = 1,500 (the connection type search ends, the network is discretized and fine-tuned), the network There will be a slight performance degradation. But it can be quickly improved to a higher level by the next stage of training. (2) It can be found that IP method brings two benefits: improved network performance and a more stable training process. The figures showed that the red solid line (mean) has always performed better than the blue solid line without the IP method; at the same time, the red shadow (standard deviation) has always been narrower than the blue shadow, which means a more stable network architecture search process.

The effect of IP rules is mainly to stabilize the performance loss caused by architecture changes when the network architecture undergoes huge changes. Therefore, we found that at 750 epoch, that is, the network plays the most significant role from searching for cell size to searching for connection type: the loss distribution with ip rules (red shading) is much smaller than the loss distribution without ip rules (blue shading). At epoch 1,500, since the fine-tuning phase does not involve drastic architectural changes, the role of IP rules is relatively limited.

### 3.5 The computational resources required for HRMAS

The proposed HRMAS bi-level optimization process is similar to DARTS, so the overall computational complexity is similar to DARTS; the IP method is an localized unsupervised learning method and does not constitute significant computational consumption. Furthermore, our proposed SC-ML topology greatly reduces the search space. Specifically, as the Figure 7 shows, the HRMAS optimization of a SC-ML layer, with *n* neurons, a motif size of *s* and *n*/*s* motifs, reduces the parameters that need to be optimized for the recurrent connection matrix from *O*(*n*^2^) to *O*(*sn*): *O*(*n*/*s*) inter-motif connections + *O*(*n*/*s* * *s*^2^) intra-motif connections + *O*(*n*) neuron hyperparameters. Generally, *s* ≪ *n*, which reduces the parameter space of recurrent connections to linear growth with the neuron numbers, allowing our algorithm scale well. Specifically, a complete training process of a RSNN with 800 neurons hidden layer for TI46-alpha dataset, including 150 epoch for cell size search, 150 epoch for connection type search and 400 epoch for finetune, takes 4 h on single NVIDIA GeForce RTX 3090 GPU.

## 4 Conclusion

We present an RSNN architecture based on SC-ML layers composed of multiple recurrent motifs with sparse inter-motif connections as a solution to constructing large recurrent spiking neural models. We further propose the automated architectural optimization framework HRMAS hybridizing the “evolution” of the architectural parameters and corresponding synaptic weights based on backpropagation and biologically-inspired mitigation of risks of architectural changes using intrinsic plasticity. We show that HRMAS-optimized RSNNs impressively improve performance on four datasets over the previously reported state-of-the-art RSNNs and SNNs. Notably, our HRMAS framework can be easily extended to more flexible network architectures, optimizing sparse and scalable RSNN architectures. By sharing the PyTorch implementation of our HRMAS framework, this work aims to foster advancements in high-performance RSNNs for both general-purpose and dedicated neuromorphic computing platforms, potentially inspiring innovative designs in brain-inspired recurrent spiking neural models and their energy-efficient deployment.

## Data availability statement

The original contributions presented in the study are included in the article/supplementary material, further inquiries can be directed to the corresponding author.

## Author contributions

WZ: Writing – original draft, Writing – review & editing. HG: Writing – original draft, Writing – review & editing. PL: Writing – original draft, Writing – review & editing.

## References

[B1] Amir A. Taba B. Berg D. Melano T. McKinstry J. Di Nolfo M.. (2017). A low power, fully event-based gesture recognition system, in Proceedings of the IEEE Conference on Computer Vision and Pattern Recognition (Honolulu, HI: IEEE), 7243–7252. 10.1109/CVPR.2017.781

[B2] Anumula J. Neil D. Delbruck T. Liu S.-C. (2018). Feature representations for neuromorphic audio spike streams. Front. Neurosci. 12:23. 10.3389/fnins.2018.00023 29479300 PMC5811520

[B3] Baddeley R. Abbott L. F. Booth M. C. Sengpiel F. Freeman T. Wakeman E. A.. (1997). Responses of neurons in primary and inferior temporal visual cortices to natural scenes. Proc. R. Soc. London B Biol. Sci. 264, 1775–1783. 10.1098/rspb.1997.0246 9447735 PMC1688734

[B4] Bellec G. Salaj D. Subramoney A. Legenstein R. Maass W. (2018). Long short-term memory and learning-to-learn in networks of spiking neurons, in 32nd Conference on Neural Information Processing Systems (NeurIPS 2018) (Montreal, QC), 787–797.

[B5] Buzsaki G. (2006). Rhythms of the Brain. Oxford: Oxford University Press. 10.1093/acprof:oso/9780195301069.001.0001

[B6] Chakraborty B. Mukhopadhyay S. (2023). Heterogeneous recurrent spiking neural network for spatio-temporal classification. Front. Neurosci. 17:994517. 10.3389/fnins.2023.994517 36793542 PMC9922697

[B7] Chen L. Li X. Zhu Y. Wang H. Li J. Liu Y.. (2023). Intralayer-connected spiking neural network with hybrid training using backpropagation and probabilistic spike-timing dependent plasticity. Int. J. Intell. Syst. 2023. 10.1155/2023/3135668

[B8] Cho K. van Merrienboer B. Gülçehre Ç. Bahdanau D. Bougares F. Schwenk H.. (2014). Learning phrase representations using RNN encoder-decoder for statistical machine translation, in Proceedings of the 2014 Conference on Empirical Methods in Natural Language Processing (EMNLP) (Doha: Association for Computational Linguistics), 1724–1734. 10.3115/v1/D14-1179

[B9] Desai N. S. Rutherford L. C. Turrigiano G. G. (1999). Plasticity in the intrinsic excitability of cortical pyramidal neurons. Nat. Neurosci. 2:515. 10.1038/9165 10448215

[B10] Elsken T. Metzen J. H. Hutter F. (2019). Neural architecture search: a survey. J. Mach. Learn. Res. 20, 1–21. 10.1007/978-3-030-05318-5_11

[B11] Fourati R. Ammar B. Jin Y. Alimi A. M. (2020). EEG feature learning with intrinsic plasticity based deep echo state network, in 2020 international joint conference on neural networks (IJCNN) (Glasgow: IEEE), 1–8. 10.1109/IJCNN48605.2020.9207464

[B12] Gerstner W. Kistler W. M. (2002). Spiking Neuron Models: Single Neurons, Populations, Plasticity. Cambridge: Cambridge University Press. 10.1017/CBO9780511815706

[B13] Graves A. Mohamed A.-r. Hinton G. (2013). Speech recognition with deep recurrent neural networks, in 2013 IEEE international conference on acoustics, speech and signal processing (Vancouver, BC: IEEE), 6645–6649. 10.1109/ICASSP.2013.6638947

[B14] He K. Zhang X. Ren S. Sun J. (2015). Delving deep into rectifiers: surpassing human-level performance on imagenet classification, in Proceedings of the IEEE international conference on computer vision (Santiago: IEEE), 1026–1034. 10.1109/ICCV.2015.123

[B15] He W. Wu Y. Deng L. Li G. Wang H. Tian Y.. (2020). Comparing snns and rnns on neuromorphic vision datasets: similarities and differences. Neural Netw. 132, 108–120. 10.1016/j.neunet.2020.08.001 32866745

[B16] Hochreiter S. Schmidhuber J. (1997). Long short-term memory. Neural Comput. 9, 1735–1780. 10.1162/neco.1997.9.8.1735 9377276

[B17] Jaeger H. (2001). The echo state approach to analysing and training recurrent neural networks-with an erratum note. National Research Center for Information Technology GMD Technical Report, 148. Bonn: German, 13.

[B18] Jin Y. Zhang W. Li P. (2018). Hybrid macro/micro level backpropagation for training deep spiking neural networks. Adv. Neural Inf. Process. Syst. 31, 7005–7015. 10.48550/arXiv.1805.07866

[B19] Kim Y. Li Y. Park H. Venkatesha Y. Panda P. (2022). Neural architecture search for spiking neural networks, in Computer Vision ECCV 2022: 17th European Conference, Tel Aviv, Israel, October 23–27, 2022, Proceedings, Part XXIV (Berlin: Springer). 10.1007/978-3-031-20053-3_3

[B20] Kingma D. P. Ba J. (2014). Adam: a method for stochastic optimization. arXiv [Preprint]. arXiv:1412.6980. 10.48550/arXiv.1412.6980

[B21] Ko H. Hofer S. B. Pichler B. Buchanan K. A. Sjöström P. J. Mrsic-Flogel T. D. (2011). Functional specificity of local synaptic connections in neocortical networks. Nature 473, 87–91. 10.1038/nature09880 21478872 PMC3089591

[B22] Lazar A. Pipa G. Triesch J. (2007). Fading memory and time series prediction in recurrent networks with different forms of plasticity. Neural Netw. 20, 312–322. 10.1016/j.neunet.2007.04.020 17556114

[B23] Liberman M. Amsler R. Church K. Fox E. Hafner C. Klavans J.. (1991). TI 46-word LDC93S9. Available online at: https://catalog.ldc.upenn.edu/LDC93S9

[B24] Liu H. Simonyan K. Yang Y. (2018). Darts: differentiable architecture search, in International Conference on Learning Representations (New Orleans, LA).

[B25] Maass W. (1997). Networks of spiking neurons: the third generation of neural network models. Neural Netw. 10, 1659–1671. 10.1016/S0893-6080(97)00011-7

[B26] Maass W. Natschläger T. Markram H. (2002). Real-time computing without stable states: a new framework for neural computation based on perturbations. Neural Comput. 14, 2531–2560. 10.1162/089976602760407955 12433288

[B27] Maes A. Barahona M. Clopath C. (2020). Learning spatiotemporal signals using a recurrent spiking network that discretizes time. PLoS Comput. Biol. 16:e1007606. 10.1371/journal.pcbi.1007606 31961853 PMC7028299

[B28] Maffei A. Fontanini A. (2009). Network homeostasis: a matter of coordination. Curr. Opin. Neurobiol. 19, 168–173. 10.1016/j.conb.2009.05.012 19540746 PMC3427905

[B29] Marder E. Abbott L. Turrigiano G. G. Liu Z. Golowasch J. (1996). Memory from the dynamics of intrinsic membrane currents. Proc. Nat. Acad. Sci. 93, 13481–13486. 10.1073/pnas.93.24.13481 8942960 PMC33634

[B30] Na B. Mok J. Park S. Lee D. Choe H. Yoon S.. (2022). AutoSNN: towards energy-efficient spiking neural networks, in Proceedings of the 39th International Conference on Machine Learning (Baltimore, MD: PMLR), 162.

[B31] Neftci E. O. Mostafa H. Zenke F. (2019). Surrogate gradient learning in spiking neural networks: bringing the power of gradient-based optimization to spiking neural networks. IEEE Signal Process. Mag. 36, 51–63. 10.1109/MSP.2019.2931595

[B32] Orchard G. Jayawant A. Cohen G. K. Thakor N. (2015). Converting static image datasets to spiking neuromorphic datasets using saccades. Front. Neurosci. 9:437. 10.3389/fnins.2015.00437 26635513 PMC4644806

[B33] Pan W. Zhao F. Han B. Dong Y. Zeng Y. (2024). Emergence of brain-inspired small-world spiking neural network through neuroevolution. iScience 27:108845. 10.1016/j.isci.2024.108845 38327781 PMC10847652

[B34] Perin R. Berger T. K. Markram H. (2011). A synaptic organizing principle for cortical neuronal groups. Proc. Nat. Acad. Sci. 108, 5419–5424. 10.1073/pnas.1016051108 21383177 PMC3069183

[B35] Real E. Aggarwal A. Huang Y. Le Q. V. (2019). Regularized evolution for image classifier architecture search. Proc. AAAI Conf. Artif. Intell. 33, 4780–4789. 10.1609/aaai.v33i01.33014780

[B36] Seeman S. C. Campagnola L. Davoudian P. A. Hoggarth A. Hage T. A. Bosma-Moody A.. (2018). Sparse recurrent excitatory connectivity in the microcircuit of the adult mouse and human cortex. Elife 7:e37349. 10.7554/eLife.37349.032 30256194 PMC6158007

[B37] Shrestha S. B. Orchard G. (2018). Slayer: spike layer error reassignment in time, in 32nd Conference on Neural Information Processing Systems (NeurIPS 2018) (Montréal, QC), 1412–1421.

[B38] Srinivasan G. Panda P. Roy K. (2018). Spilinc: spiking liquid-ensemble computing for unsupervised speech and image recognition. Front. Neurosci. 12:524. 10.3389/fnins.2018.00524 30190670 PMC6116788

[B39] Tian S. Qu L. Wang L. Hu K. Li N. Xu W.. (2021). A neural architecture search based framework for liquid state machine design. Neurocomputing 443, 174–182. 10.1016/j.neucom.2021.02.076

[B40] Tien N.-W. Kerschensteiner D. (2018). Homeostatic plasticity in neural development. Neural Dev., 13, 1–7. 10.1186/s13064-018-0105-x 29855353 PMC5984303

[B41] Voelker A. Kajić I. Eliasmith C. (2019). Legendre memory units: continuous-time representation in recurrent neural networks, in 33rd Conference on Neural Information Processing Systems (NeurIPS 2019) (Vancouver, BC), 15570–15579.

[B42] Wang Q. Li P. (2016). D-lsm: deep liquid state machine with unsupervised recurrent reservoir tuning, in 2016 23rd International Conference on Pattern Recognition (ICPR) (Cancun: IEEE), 2652–2657. 10.1109/ICPR.2016.7900035

[B43] Wijesinghe P. Srinivasan G. Panda P. Roy K. (2019). Analysis of liquid ensembles for enhancing the performance and accuracy of liquid state machines. Front. Neurosci. 13:504. 10.3389/fnins.2019.00504 31191219 PMC6546930

[B44] Wistuba M. Rawat A. Pedapati T. (2019). A survey on neural architecture search. arXiv [Preprint]. arXiv:1905.01392. 10.48550/arXiv.1905.01392

[B45] Wu Y. Deng L. Li G. Zhu J. Shi L. (2018). Spatio-temporal backpropagation for training high-performance spiking neural networks. Front. Neurosci. 12:331. 10.3389/fnins.2018.00331 29875621 PMC5974215

[B46] Zela A. Elsken T. Saikia T. Marrakchi Y. Brox T. Hutter F.. (2019). Understanding and robustifying differentiable architecture search, in International Conference on Learning Representations (Addis Ababa: ICLR).

[B47] Zhang A. Gao Y. Niu Y. Li X. Chen Q. (2020). Intrinsic plasticity for online unsupervised learning based on soft-reset spiking neuron model. IEEE Trans. Cogn. Dev. Syst. 15, 337–347. 10.1109/TCDS.2020.3041610

[B48] Zhang A. Li X. Gao Y. Niu Y. (2021). Event-driven intrinsic plasticity for spiking convolutional neural networks. IEEE Trans. Neural Netw. Learn. Syst. 33, 1986–1995. 10.1109/TNNLS.2021.3084955 34106868

[B49] Zhang A. Zhou H. Li X. Zhu W. (2019). Fast and robust learning in spiking feed-forward neural networks based on intrinsic plasticity mechanism. Neurocomputing 365, 102–112. 10.1016/j.neucom.2019.07.00927534393

[B50] Zhang W. Li P. (2019b). Spike-train level backpropagation for training deep recurrent spiking neural networks, in Advances in Neural Information Processing Systems (NeurIPS 2019) (Vancouver, BC), 7800–7811.

[B51] Zhang W. Li P. (2020). Temporal spike sequence learning via backpropagation for deep spiking neural networks, in 34th Conference on Neural Information Processing Systems (NeurIPS 2020) (Vancouver, BC).

[B52] Zhang W. Li P. (2021a). Spiking neural networks with laterally-inhibited self-recurrent units, in 2021 International Joint Conference on Neural Networks (IJCNN) (Shenzhen: IEEE), 18. 10.1109/IJCNN52387.2021.9533726

[B53] Zhang W. Li P. (2021b). Skip-connected self-recurrent spiking neural networks with joint intrinsic parameter and synaptic weight training. Neural Comput. 33, 1886–1913. 10.1162/neco_a_01393 34411267

[B54] Zhang Y. Li P. Jin Y. Choe Y. (2015). A digital liquid state machine with biologically inspired learning and its application to speech recognition. IEEE Trans. Neural Netw. Learn. Syst. 26, 2635–2649. 10.1109/TNNLS.2015.2388544 25643415

[B55] Zhang W. Li P. (2019a). Information-theoretic intrinsic plasticity for online unsupervised learning in spiking neural networks. Front. Neurosci. 13:31. 10.3389/fnins.2019.00031 30804736 PMC6371195

[B56] Zhou Y. Jin Y. Ding J. (2020). Surrogate-assisted evolutionary search of spiking neural architectures in liquid state machines. Neurocomputing 406, 12–23. 10.1016/j.neucom.2020.04.079

[B57] Zoph B. Le Q. V. (2017). Neural architecture search with reinforcement learning, in 5th International Conference on Learning Representations (Toulon: ICLR). Available online at: https://openreview.net/forum?id=r1Ue8Hcxg

